# Supplementary material for: CDK4 phosphorylation status and a linked gene expression profile predict sensitivity to palbociclib
Source: EMBO Mol Med. 2017 May 31;9(8):1052–66. doi: 10.15252/emmm.201607084 (PMC5538335; doi:10.15252/emmm.201607084)
Supplement: Supplementary file 1 — Appendix [file EMMM-9-1052-s001.pdf]

APPENDIX

Contents :

-**Appendix Supplementary Text** including detailed methods and statistical analyses, detailed descriptions and legends to Appendix Supplementary Figures and Tables.

-**Bibliography of Appendix Supplementary Text**.

-**Appendix Supplementary Figure S1**. Distributions of the expression values of the 11 genes selected to predict the CDK4 modification profile and of the *MKI67* gene among breast tumors with different CDK4 modification profiles.

-**Appendix Supplementary Figure S2**. Statistical evaluation of the performance of the CDK4 modification prediction.

-**Appendix Supplementary Figure S3**. Relations between the tumor Ki-67 labeling index and the expression of the 11 genes selected to predict the CDK4 modification profile and of the *MKI67* gene in breast tumors with different CDK4 modification profiles

-**Appendix Supplementary Figure S4**. Relationships between the coefficients of correlation to the 3 profile-specific centroids and GGI, Oncotype DX (OnDx), Rb LOH scores or Ki-67 labeling index of breast tumors.

-**Appendix Supplementary Figure S5**. Sensitivity to PD0332991 of breast cancer cell lines is associated with CDK4 phosphorylation.

-**Appendix Supplementary Figure S6**. Distributions of the expression values of the 11 genes selected to predict the CDK4 modification profile and of the *MKI67* gene among breast cancer cell lines with different CDK4 modification profiles.

-**Appendix Supplementary Figure S7**. Heatmap of the expression values of the optimized 11 gene expression signature in breast cancer cell lines.

-**Appendix Supplementary Figure S8**. Validation of the automatic estimation of the proportion of BrdU-labelled cells.

-**Appendix Supplementary Figure S9**. Validation of Rb loss index computation.

-**Appendix Supplementary Table S1**. Sources and dilutions of antibodies used in this study.

-**Appendix Supplementary Table S2**. Studies used to evaluate Affymetrix-Agilent probe correspondence.

APPENDIX SUPPLEMENTARY TEXT

Detailed methods and statistical approaches

BrdU-incorporation image acquisition and analysis

Culture plates were imaged with a Zeiss Axio Observer.Z1 wide-field microscope equipped with a 10/0.3x EC Plan Neofluar objective and an AxioCam HSm camera. For each well, a grid of 3x3 fields (each of them 660 µm x 492 µm) was automatically defined using the Mark&Find option of the AxioVision software. DAPI signal was recorded using a 365 nm LED excitation and the Zeiss filterset 49. Alexa Fluor 488 signal was recorded using a 470 nm LED excitation and the Zeiss filterset 52. Exposure times were chosen so as to maximize the signal while avoiding saturation. They were kept constant through all images within one experiment. A software-based autofocus was performed on the DAPI channel. Images were analyzed semi-automatically with a custom-made ImageJ macro. For each field of view, two masks were created, representing the areas positive for the DAPI or for both the DAPI and the BrdU stainings, respectively. Nuclei were then segmented within these masks using the "Analyze particles" function. The total number of nuclei and the area that they represent were recorded to compute the BrdU-positive to total nuclei ratios. The segmentation of the nuclei was verified visually and only values extracted from correctly segmented images were selected for further analysis. Good correlation between the automatic counts obtained using this macro and manual counts using the Cell Counter Image J plugin was achieved (Appendix Supplementary Fig S8) except when only a few cells were stained. Each processed image was therefore systematically inspected to correct low counts and remove image with staining artefacts from the analysis. Images with less than 10 labeled nuclei were counted manually with the Image J cell counter plugin.

Microarray data analyses

Gene expression profiles from breast tumors published in GEO or Array Express (Datasets EV1 and EV8) were downloaded and summarized using the fRMA R package (1). 4034 unique breast tumor profiles were merged into a single dataset with corresponding clinical records obtained from the respective publications. Identity of the patients was defined based on the identity of the patient reference number and confirmed by correlating the gene expression profiles of putative duplicated samples. A correlation coefficient of one indicated identical samples. Since fRMA uses a common reference for correcting probe effects, two profiles from an identical patient will be identical even if included in different studies. Distribution of the patients in the different datasets

is reported in Dataset EV1. Cox survival analyses were performed in R with the Survival package using the predicted CDK4 modification profiles as stratification variable. Molecular subtypes were defined as the subtype identified on the basis of each tumor gene expression profile using the genefu package (2) and the PAM50 references (3). Genefu package was also used to define for each gene expression profile according to published clinical records the genomic grade index and the Oncotype DX risk. Chi-square test incorporated in the table function of R was used to reject the hypothesis that the data are independent. Non parametric Kruskal-Wallis test included in the R package asbio was implemented to test the null hypothesis of identical means of the distributions of values among multiple levels of biological parameters.

Probe sets analyzed in this study were first selected on the basis of the consistency between their annotation and the homology of the probes with their target sequence as reported in the Geneannot (4) or PLANdbAffy (5) databases and on the reproducibility of the expression values corresponding to common breast cancer cell lines described in the studies GSE10890, GSE12777 and GSE16795. A probe set was considered reliable when the corresponding Geneannot annotation quality markers and the specificity and sensitivity indexes were equal to one. A probe set was considered reliable when more than 63% of the probes are flagged as green (perfect match) or yellow (perfect match but with sequence in noncoding RNA) in the PLANdbAffy database. To evaluate reproducibility, we first identified breast cancer reference cell lines that are commonly described in the studies cited above. Next, the expression values for each probe set analyzed in the common cell lines in one study were linearly correlated to the corresponding values described in the two other studies. A probe set was considered reliable if the averaged Pearson correlation coefficient obtained in this way was above 0.5. Out of the 22283 probe sets common between the Affymetrix Human Genome U133A and U133plus2 arrays, 9976 met these criteria. In addition, probe sets with good inter-platform compatibility were further selected based on the correlation between gene expression measured with the Affymetrix platform and with RNA-seq (6). To identify Agilent-Affymetrix compatible probes, the probe sets of both platforms were matched with their corresponding Entrez gene ID before the relative expression values determined with both platforms in at least 20 common samples of cell lines were compared by Spearman correlation. The studies used for these comparisons are listed in the Appendix Supplementary Table S2. Agilent and Affymetrix probe sets are considered compatible when the correlation coefficient is at least above 0.5. Lists of probe sets with increasing compatibility were selected with correlation coefficients of 0.6, 0.7, 0.75 and 0.8.

Development of a gene expression-based predictor of CDK4 modification profiles.

Probe sets differentially expressed in tumor samples with different CDK4 modification profiles were selected using the sam function of the samr package with either the "Multiclass" or the "Two classes unpaired" response

types when the three types of CDK4 profiles or binary classifications (A/non A, H/non H or L/non L) were considered, respectively. The probe set with the minimum q-value statistic having a positive or negative correlation with the outcome were selected. If less than 20 probe sets met these criteria, the 20 first probe sets negatively or positively correlated with the outcome were recorded. Alternatively, the pROC package was also used to select probe sets that could differentiate samples with different CDK4 modification profiles. To this end, a binary category (A/non A, H/non H or L/non L) was first attributed to each sample. Second, for each probe set, a Receiver Operating Characteristic (ROC) curve was built and its area under the curve value (AUC) extracted using the roc and auc functions of the pROC package, respectively. Probe sets with AUC values above 0.9 or below 0.1 were selected for further analysis. When several probe sets corresponding to a same Entrez gene ID were selected, we chose the one with the highest inter-study or inter-platform correlation coefficient described above. The probe set selection was performed either on the first cohort of 19 tumors from the Bordet Institute (on 50 balanced subsets of tumors that included 4 profile A tumors, 8 profile H tumors and 5 profile L tumors as randomly selected from the 19 initial and the 33 new tumors from the Bordet Institute to avoid sampling bias), or on an extended subset of 13 samples from the first cohort merged with 26 samples of the second cohort. Probe sets in common between different lists were further used to create consensus probe set lists. Reference centroids were built with each list by computing the mean expression levels of each probes for each set of tumors with the A, H or L CDK4 modification profile, respectively. These sets corresponded to the initial exploratory set of 19 tumors analyzed at the Bordet Institute or to the same subsets that were used to identify the differentially expressed probes. The concordance rates between observed and predicted CDK4 modification profiles among the tumors of the Bordet Institute were used to rank the lists generated as described above.

Next, we used the R package pROC to shrink the 26 best probe set lists defined above to the minimal optimal probe set list that was correctly predicted the CDK4 modification profiles of the tumors analyzed at the Bordet Institute. To this end, the expression values of each probe set included in the list under evaluation were compared by Spearman correlation with the three reference centroids described above as defined for each CDK4 modification profile. The three resulting Spearman correlation coefficients were binarized to estimate three areas under the ROC curve corresponding to profile A-non A, profile H-non H and profile L-non L classifications using the roc and auc functions of the pROC package. The product of these three AUCs was used as optimization statistic. At each optimization step, one probe at a time was removed from the starting list to generate new centroids to which each tumor gene expression was compared, to binarize the corresponding Spearman correlation coefficient and to estimate the product of the three AUCs. The list of probe sets giving the highest AUC product was selected for the next round of optimization until the list contained 5 probe sets. The list with the highest AUC product was selected to evaluate the total observation/prediction or the class-specific

observation/prediction concordance rates. One list was ultimately selected as the shortest list able to correctly classify the 56 tumors coming from the Bordet Institute and University of Antwerp (84% concordance). The probes of this particular list were selected with a "Multiclass" SAM analysis of 13 samples from the first cohort of Bordet Institute merged with 26 samples from the second cohort of Bordet Institute (67% of the samples). Only probe sets from this list with good compatibility with corresponding Agilent probe sets (correlation coefficient >0.7) were considered for further development of the predictor. Centroids used to optimize the list were defined with the same 39 samples as those used to select the probe sets. To check if the performance of the optimal classifier could have occurred by chance alone, the performance of the optimal classifier was compared to the distribution of the performance achieved with 1000 random lists of 11 genes or with random 1000 random permutations of the patient labels. This was neither the case when 11 genes were randomly chosen nor when the patient labels were permutated (Appendix Supplementary Fig S2).

Definition of the Rb loss index and of the Rb LOH score.

Knudsen's lab introduced a Rb loss signature of 159 genes based on differential gene expression profiles definition using mouse fibroblasts or hepatocytes from Rbfl/fl mice with or without cre excision of Rb (7). The signature was analyzed in a merged cohort of breast tumors including the studies E-TABM158, GSE1456, GSE6532, GSE7390, GSE9195 and GSE12093. The signature index was built by taking the average of the median-centered probes for each sample analyzed. When multiple probe sets correspond to a single gene, the average expression value scaled by the largest SD was used. The 159 genes of the signature published in the supplemental material of Ertel's publication (7) were mapped to their corresponding Affymetrix probe sets via their Entrez gene ID. When multiple probe sets corresponded to the same gene ID, we used only the probe set with the highest inter-study correlation as described above. Compared to the published values, the Rb loss index computed based on the fRMA summarized expression values of the breast tumors samples of the E-TABM158, GSE1456, GSE6532, GSE7390, GSE9195 and GSE12093 studies correlated well with the values of Ertel's publication (7) except for the clearly off-setted values of the E-TABM158 study (Appendix Supplementary Fig S9A). Therefore, this study was discarded to compute the median used to center each probe set expression values. A better correlation between computed and published Rb loss index was achieved when only the samples from the GSE1456, GSE6532, GSE7390, GSE9195 and GSE12093 studies were used to compute the medians used to center the data (Appendix Supplementary Fig S9B). These medians were thereafter used as reference to center the expression values of novel samples.

By implementing a sam analysis pipeline, the group of Perou identified a set of 354 genes differentially expressed between 67 informative cases out of a cohort of 88 breast tumors with or without RB1 loss of

heterozygosity (8). The latter was defined based on the detection of two polymorphic marker (a variable number tandem repeat in intron 20 and D13S153, a microsatellite marker for RB1 LOH analyses). LOH was called when there was at least a 50% loss of an allele/band in the tumor for at least one of the two polymorphic markers compared to normal DNA isolated from lymphocytes or normal adjacent tissue. We successfully mapped 297 of the 339 genes selected in Perou's group work (9) to Affymetrix probe sets. 293 probe sets corresponding to unique Entrez gene IDs were further selected. The Rb LOH score was defined as the median of the median-centered gene expression measured by these 293 probe sets. The median values used to center the data are the medians of each selected probe among the 4034 published Affymetrix gene expression profiles of breast tumors.

Characteristics of the 11 selected probes.

Several of these 11 selected probes detect the expression of key genes implicated in the execution of the cell cycle in addition to *CCNE1* and *CDKN2A*. The protein encoded by *CCDC99* also named *SPDL1* is involved in the localization of dynein and dynactin to the mitotic kinetochore (10). *NUP155* protein is a nucleoporin important for the generation of the nuclear envelope (11, 12). *TAGLN2* is often overexpressed in gastric cancer (13) and hepatocarcinoma (14) and is thought to be involved in proliferation and migration (15). *FBXL5* protein is a targeting subunit of a SKP1-cullin-F-box ubiquitin ligase complex regulating the degradation of, among others, the human single-strand (ss) DNA binding protein 1 (hSSB1) known to participate in DNA damage response and maintenance of genome stability by regulating the initiation of ATM-dependent signaling (16) and to modulate the DNA binding and stability of the EMT inducer Snail1 (17). *TIMM17A* is overexpressed in breast cancer and its protein contributes to a pre-protein import complex, essential for mitochondrial function (18). The other probe sets detect the expression of genes playing key roles in cellular signaling. Rab31 is a small GTP binding protein controlling vesicles and granule traffic leading to a switch from an invasive to a proliferative phenotype in breast cancer (19). It plays a role in the control of plasma membrane localization of EGFR (20). *PPP1R3C* protein is a protein phosphatase 1 regulatory subunit binding to glycogen. Its expression is increased by hypoxia (21). The gelsolin encoded by *GSN* is an actin-binding protein often lost in breast cancer (22-24). *TP53TG1* encodes a long non coding RNA induced in a wild-type TP53-dependent manner by conditions of cellular stress (ultraviolet irradiation or exposure to bleomycin or cisplatin) (25).

Bibliography of Appendix Supplementary Text.

(1) McCall MN, Bolstad BM, Irizarry RA. Frozen robust multiarray analysis (fRMA). Biostatistics 2010;11:242-53.

(2) Gendoo DM, Ratanasirigulchai N, Schroder MS, Pare L, Parker JS, Prat A, et al. Genefu: an R/Bioconductor package for computation of gene expression-based signatures in breast cancer. Bioinformatics 2015.

(3) Parker JS, Mullins M, Cheang MC, Leung S, Voduc D, Vickery T, et al. Supervised risk predictor of breast cancer based on intrinsic subtypes. J Clin Oncol 2009;27:1160-7.

(4) Ferrari F, Bortoluzzi S, Coppe A, Sirota A, Safran M, Shmoish M, et al. Novel definition files for human GeneChips based on GeneAnnot. BMC Bioinformatics 2007;8:446.

(5) Nurtdinov RN, Vasiliev MO, Ershova AS, Lossev IS, Karyagina AS. PLANdbAffy: probe-level annotation database for Affymetrix expression microarrays. Nucleic Acids Res 2010;38:D726-D730.

(6) Fumagalli D, Blanchet-Cohen A, Brown D, Desmedt C, Gacquer D, Michiels S, et al. Transfer of clinically relevant gene expression signatures in breast cancer: from Affymetrix microarray to Illumina RNA-Sequencing technology. BMC Genomics 2014;15:1008.

(7) Ertel A, Dean JL, Rui H, Liu C, Witkiewicz AK, Knudsen KE, et al. RB-pathway disruption in breast cancer: differential association with disease subtypes, disease-specific prognosis and therapeutic response. Cell Cycle 2010;9:4153-63.

(8) Herschkowitz JI, He X, Fan C, Perou CM. The functional loss of the retinoblastoma tumour suppressor is a common event in basal-like and luminal B breast carcinomas. Breast Cancer Res 2008;10:R75.

(9) Gatza ML, Silva GO, Parker JS, Fan C, Perou CM. An integrated genomics approach identifies drivers of proliferation in luminal-subtype human breast cancer. Nat Genet 2014;46:1051-9.

(10) Barisic M, Sohm B, Mikolcevic P, Wandke C, Rauch V, Ringer T, et al. Spindly/CCDC99 is required for efficient chromosome congression and mitotic checkpoint regulation. Mol Biol Cell 2010;21:1968-81.

(11) Hawryluk-Gara LA, Shibuya EK, Wozniak RW. Vertebrate Nup53 interacts with the nuclear lamina and is required for the assembly of a Nup93-containing complex. Mol Biol Cell 2005;16:2382-94.

(12) Busayavalasa K, Chen X, Farrants AK, Wagner N, Sabri N. The Nup155-mediated organisation of inner nuclear membrane proteins is independent of Nup155 anchoring to the metazoan nuclear pore complex. J Cell Sci 2012;125:4214-8.

(13) Elsner M, Rauser S, Maier S, Schone C, Balluff B, Meding S, et al. MALDI imaging mass spectrometry reveals COX7A2, TAGLN2 and S100-A10 as novel prognostic markers in Barrett's adenocarcinoma. J Proteomics 2012;75:4693-704.

(14) Zhang Y, Ye Y, Shen D, Jiang K, Zhang H, Sun W, et al. Identification of transgelin-2 as a biomarker of colorectal cancer by laser capture microdissection and quantitative proteome analysis. Cancer Sci 2010;101:523-9.

(15) Leung WK, Ching AK, Chan AW, Poon TC, Mian H, Wong AS, et al. A novel interplay between oncogenic PFTK1 protein kinase and tumor suppressor TAGLN2 in the control of liver cancer cell motility. Oncogene 2011;30:4464-75.

(16) Chen ZW, Liu B, Tang NW, Xu YH, Ye XY, Li ZM, et al. FBXL5-mediated degradation of single-stranded DNA-binding protein hSSB1 controls DNA damage response. Nucleic Acids Res 2014;42:11560-9.

(17) Vinas-Castells R, Frias A, Robles-Lanuza E, Zhang K, Longmore GD, Garcia de HA, et al. Nuclear ubiquitination by FBXL5 modulates Snail1 DNA binding and stability. Nucleic Acids Res 2014;42:1079-94.

(18) Salhab M, Patani N, Jiang W, Mokbel K. High TIMM17A expression is associated with adverse pathological and clinical outcomes in human breast cancer. Breast Cancer 2012;19:153-60.

(19) Grismayer B, Solch S, Seubert B, Kirchner T, Schafer S, Baretton G, et al. Rab31 expression levels modulate tumor-relevant characteristics of breast cancer cells. Mol Cancer 2012;11:62.

(20) Chua CE, Tang BL. Engagement of the small GTPase Rab31 protein and its effector, early endosome antigen 1, is important for trafficking of the ligand-bound epidermal growth factor receptor from the early to the late endosome. J Biol Chem 2014;289:12375-89.

(21) Shen GM, Zhang FL, Liu XL, Zhang JW. Hypoxia-inducible factor 1-mediated regulation of PPP1R3C promotes glycogen accumulation in human MCF-7 cells under hypoxia. FEBS Lett 2010;584:4366-72.

(22) Asch HL, Head K, Dong Y, Natoli F, Winston JS, Connolly JL, et al. Widespread loss of gelsolin in breast cancers of humans, mice, and rats. Cancer Res 1996;56:4841-5.

(23) Dong Y, Asch HL, Medina D, Ip C, Ip M, Guzman R, et al. Concurrent deregulation of gelsolin and cyclin D1 in the majority of human and rodent breast cancers. Int J Cancer 1999;81:930-8.

(24) Thor AD, Edgerton SM, Liu S, Moore DH, Kwiatkowski DJ. Gelsolin as a negative prognostic factor and effector of motility in erbB-2-positive epidermal growth factor receptor-positive breast cancers. Clin Cancer Res 2001;7:2415-24.

(25) Takei Y, Ishikawa S, Tokino T, Muto T, Nakamura Y. Isolation of a novel TP53 target gene from a colon cancer cell line carrying a highly regulated wild-type TP53 expression system. Genes Chromosomes Cancer 1998;23:1-9.

Legends to Appendix Supplementary Figures and Tables

Appendix Supplementary Figure S1 - Distributions of the expression values of the 11 genes selected to predict the CDK4 modification profile and of the *MKI67* gene among breast tumors with different CDK4 modification profiles.

The gene expression levels of the 11 genes used to predict tumor CDK4 modification profile were measured with the Affymetrix HG-U133 plus2 platform using the indicated probe sets. The first line below the plot indicates the number of observations. The second line reports the statistical decision whether the true effect of CDK4 modification profile is significant (levels with the same letter are not significantly different at alpha = 0.05). The third and fourth lines report the respective mean and SD. The last line provides the p-value of rejection of the null hypothesis that all means are equal. Pairwise comparisons were performed with the Kruskal-Wallis test (level of confidence set at 0.95).

Appendix Supplementary Figure S2 - Statistical evaluation of the performance of the CDK4 modification prediction.

A The performance of CDK4 modification profile prediction using the optimal set of 11 probes (dotted line) was compared to the distribution of the performances of 1000 random lists of 11 probes.

B The performance of CDK4 modification profile prediction using the optimal set of 11 probes (dotted line) in the whole Bordet breast cancer sample cohort was compared to the distribution of the corresponding performances achieved when the patient labels were randomly switched 1000 times.

Appendix Supplementary Figure S3 - Correlations of mRNA levels of 11 genes used to predict the CDK4 modification profile with the corresponding Ki-67 labeling index of the tumors.

The gene expression levels of the 11 genes used to predict tumor CDK4 modification profile were measured with the Affymetrix HG-U133 plus2 platform using the indicated probe sets. These values are expressed in function of the Ki-67 labeling index measured after Ki-67 staining of parallel FFPE sections. The slope, intercept and R-square values were obtained in R by fitting a linear model (x~y) to the data. The p-value indicated is the p-value of the test for association between paired samples using the Pearson's product moment correlation coefficient obtained in R

Appendix Supplementary Figure S4 - Relationships between the coefficients of correlation to the 3 profile-specific centroids and GGI, Oncotype DX (OnDX), Rb LOH scores or Ki-67 labeling index of breast tumors.

The gene expression levels of the 11 genes used to predict tumor CDK4 modification profile were measured for each tumor with the Affymetrix HG-U133 plus2 platform using the probe sets indicated in Dataset EV6. These values were compared by Spearman correlation to three reference centroids representative of each three tumor CDK4 modification profiles. These centroids were built by computing the mean of each selected genes among each gene expression profiles representative of each three CDK4 modification profiles. The three profile-specific Spearman correlation coefficients were compared to the Genomic Grade Index (GGI) and Oncotype DX computed using the genefu package in R. They were also compared to the Rb LOH score defined in Perou's lab (8) and to the Ki-67 labeling index determined on parallel FFPE sections of the tumors stained with the DAKO Ki-67 antibody. The slope, intercept and R-square values were obtained in R by fitting a linear model (x~y) to the data. The p-value indicated is the p-value of the test for association between paired samples using the Pearson's product moment correlation coefficient obtained in R.

Appendix Supplementary Figure S5 - Sensitivity to PD0332991 of breast cancer cell lines is associated with CDK4 phosphorylation.

The effect of 24-hour treatment with increasing concentrations of PD0332991 on DNA synthesis rate was determined by BrdU labeling of the indicated cell lines seeded in 96-well plates. Relative proportion of BrdU-labeled cells is expressed as % of the mean value of untreated control cells. Error bars correspond to standard deviations scaled relative to the mean value of untreated control cells. These values were compared to cell growth estimated after 6 days with the sulforhodamine assay or after 48 hours with MTT assay using increasing concentrations of puromycine as a positive control of cytotoxicity. The 2D-gel electrophoresis profile of total protein extracts of the corresponding cell lines revealed with an anti-CDK4 antibody are also shown. The position of the main T172-phosphorylated form of CDK4 (spot 3) is circled.

A HCC1954, HCC1806, MDAMB361 and MDAMB231 cells.

B MDAMB436, MDAMB468, SKBR3 and MCF7 cells.

C BT549, HCC70, ZR75.1 and MDAMB134VI cells.

D HCC1569, HCC1937, BT474 and HCC202 cells.

E HCC38, HCC1500, BT20 and T47D cells.

Appendix Supplementary Figure S6 - Distributions of the expression values of the 11 genes selected to predict the CDK4 modification profile and of the *MKI67* gene among breast cancer cell lines with different CDK4 modification profiles.

The gene expression levels of the 11 genes used to predict tumor CDK4 modification profile were measured for each cell line with the Affymetrix HG-U133 plus2 platform using the probe sets indicated in Dataset EV6. These values were compared by Spearman correlation to three reference centroids representative of each three tumor CDK4 modification profiles. These centroids were built by computing the mean of each selected genes among each gene expression profiles representative of each 3 CDK4 modification profiles. The first line below the plot indicates the number of observations. The second lines defines the CDK4 modification profile types. The third line reports the statistical decision whether the true effect of CDK4 modification profile is significant (levels with the same letter are not significantly different at alpha = 0.05). The fourth and fifth lines report the respective mean and SD. The last line provides the p-value of rejection of the null hypothesis that all means are equal. Pairwise comparisons were performed with the Kruskal-Wallis test (level of confidence set at 0.95).

Appendix Supplementary Figure S7 - Heatmap of the expression values of the optimized 11 gene expression signature in breast cancer cell lines.

RNA was extracted in parallel to proteins from the indicated fresh frozen cell lines samples and quantified with the Affymetrix HG-U133plus 2 platform. Heatmaps were drawn with the heatmap.plus R package using the normalized expression values of the optimal 11 probe sets predicting the CDK4 modification profiles corresponding to the 56 breast tumors analyzed in this study. Displayed above each heatmap are the Rb loss index computed according to the method reported by Knudsen's lab (7), the molecular subtype defined with the genefu package based on each sample PAM50 expression value, the observed and predicted CDK4 modification profiles, and the matches between them.

Appendix Supplementary Figure S8 - Validation of the automatic estimation of the proportion of BrdU-labelled cells.

T47D and MCF7 cells were seeded in triplicate in 96-well plate at a density of 2 10^4 cells per well and cultured for two days in the presence of increasing concentrations of PD0332991. One hour before fixation with methanol, BrdU was added to the cells. The proportion of double stained cells was determined in 9 pictures per well taken with a Zeiss Axio Observer.Z1 wide-field microscope equipped with a 10/0.3x EC Plan Neofluar

objective and an AxioCam HSm camera either by manual counting using the ImageJ Cell Counter plugin or using a custom ImageJ script described in the Supplementary methods section. Proportions of double stained cells defined by the semi-automatic script are plotted against the corresponding proportions determined by manual counting. Plotted values are the average of the proportions determined manually or semi-automatically in the 9 pictures of three triplicate wells. Error bars correspond to standard deviations.

Appendix Supplementary Figure S9 - Validation of Rb loss index computation.

Correlation between the published and computed Rb loss index is illustrated with all samples used in Ertel et al publication (7) (A) or with all these samples except the ones from the E-TABM-158 study (B).

Appendix Supplementary Table S1 – Sources and dilutions of antibodies used in this study.

Appendix Supplementary Table S2 - Studies used to evaluate Affymetrix-Agilent probe correspondence.

The table details for each study the platform used, its brand, the source of information, the author of the study, and the count of cell lines analyzed that are common to the study indicated by the column headers and the first column of each row.

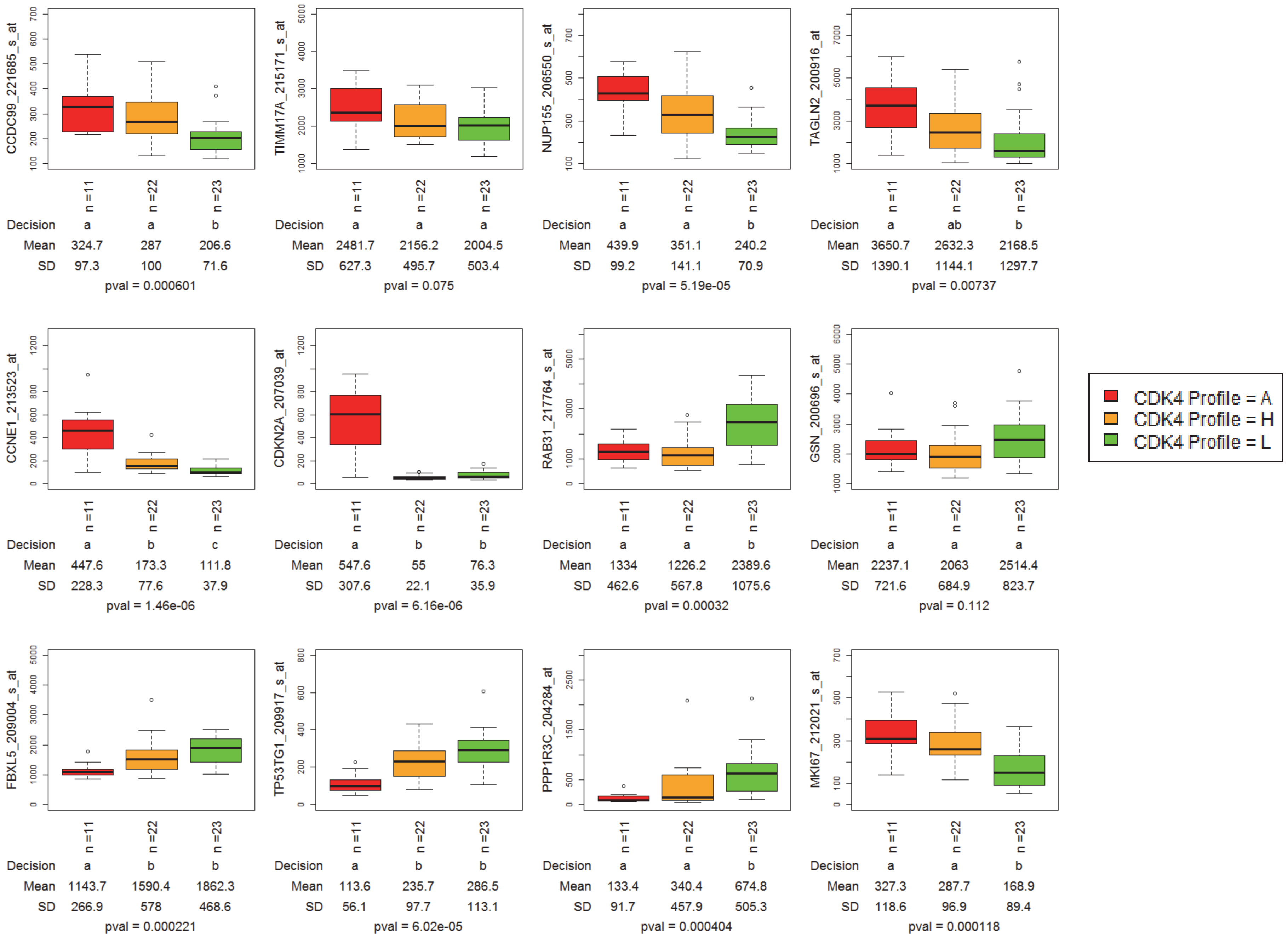

Appendix Figure S1

Doted line = best list

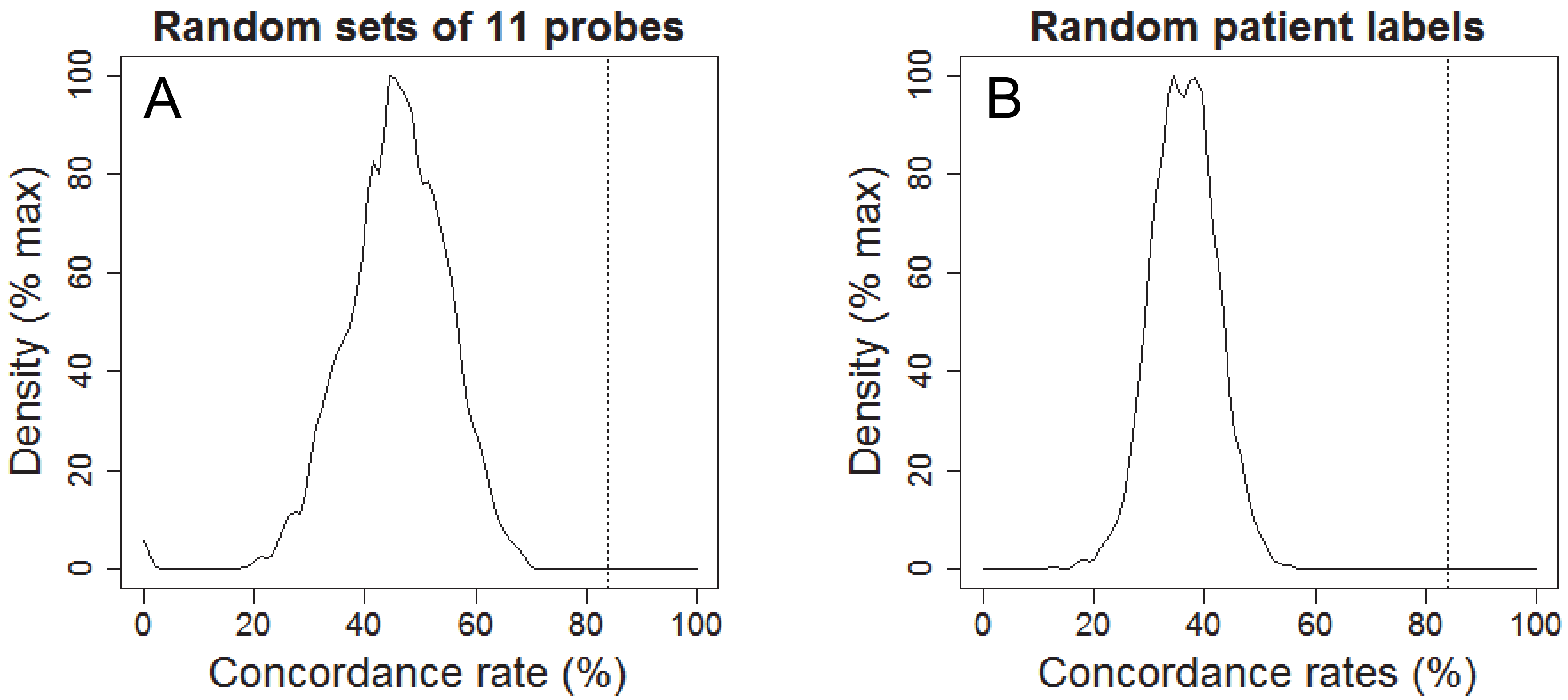

Appendix Figure S2

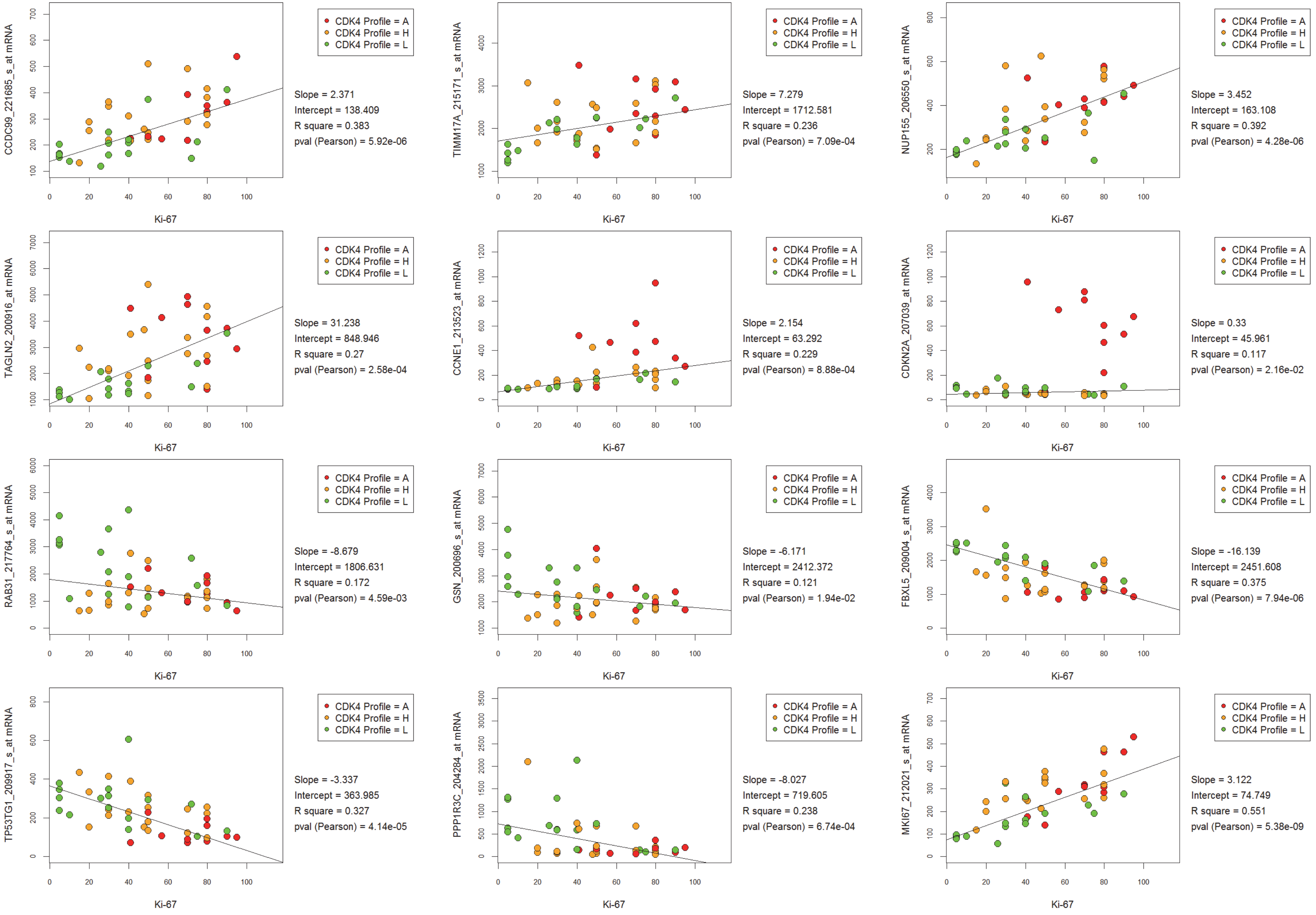

Appendix Figure S3

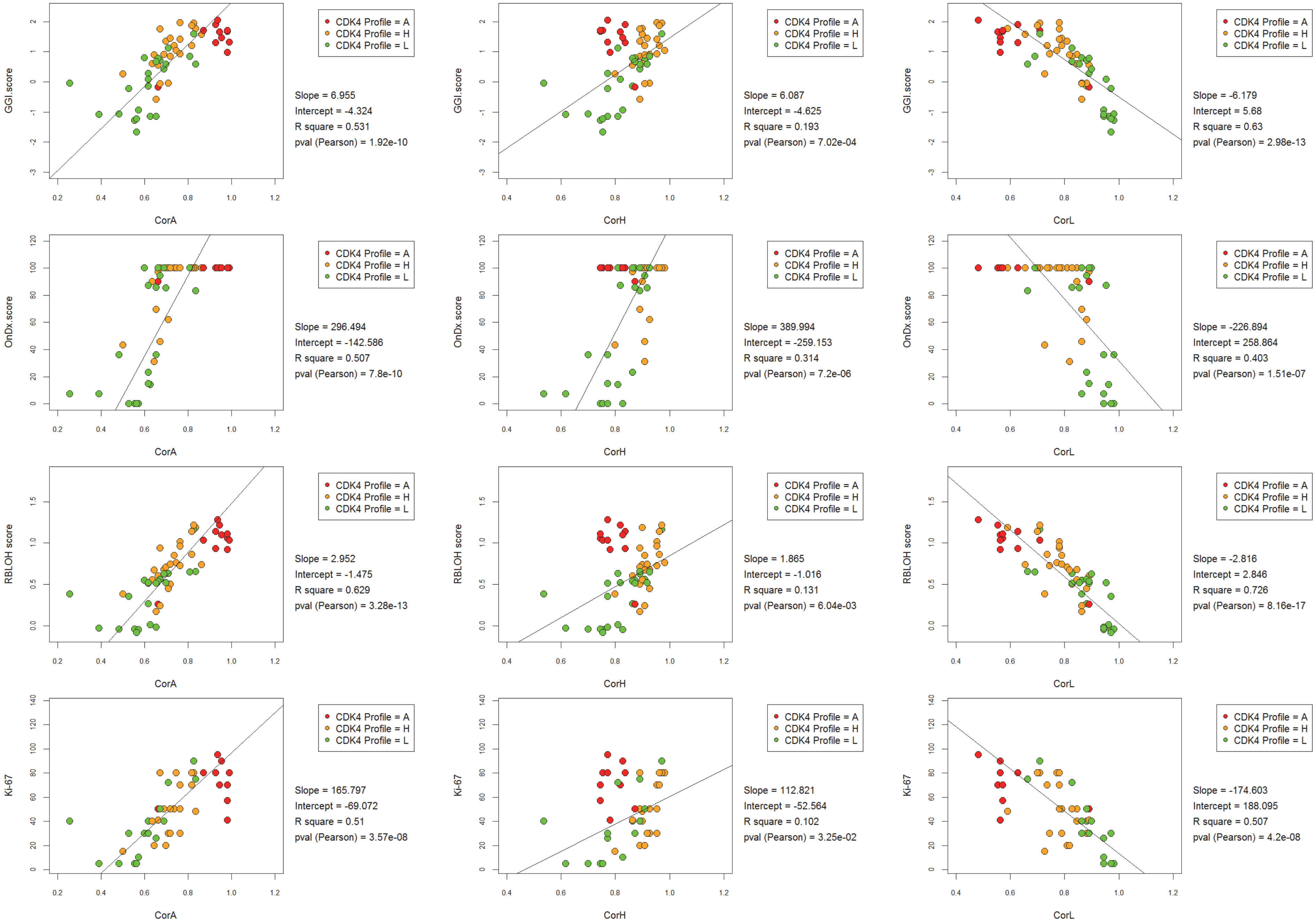

Appendix Figure S4

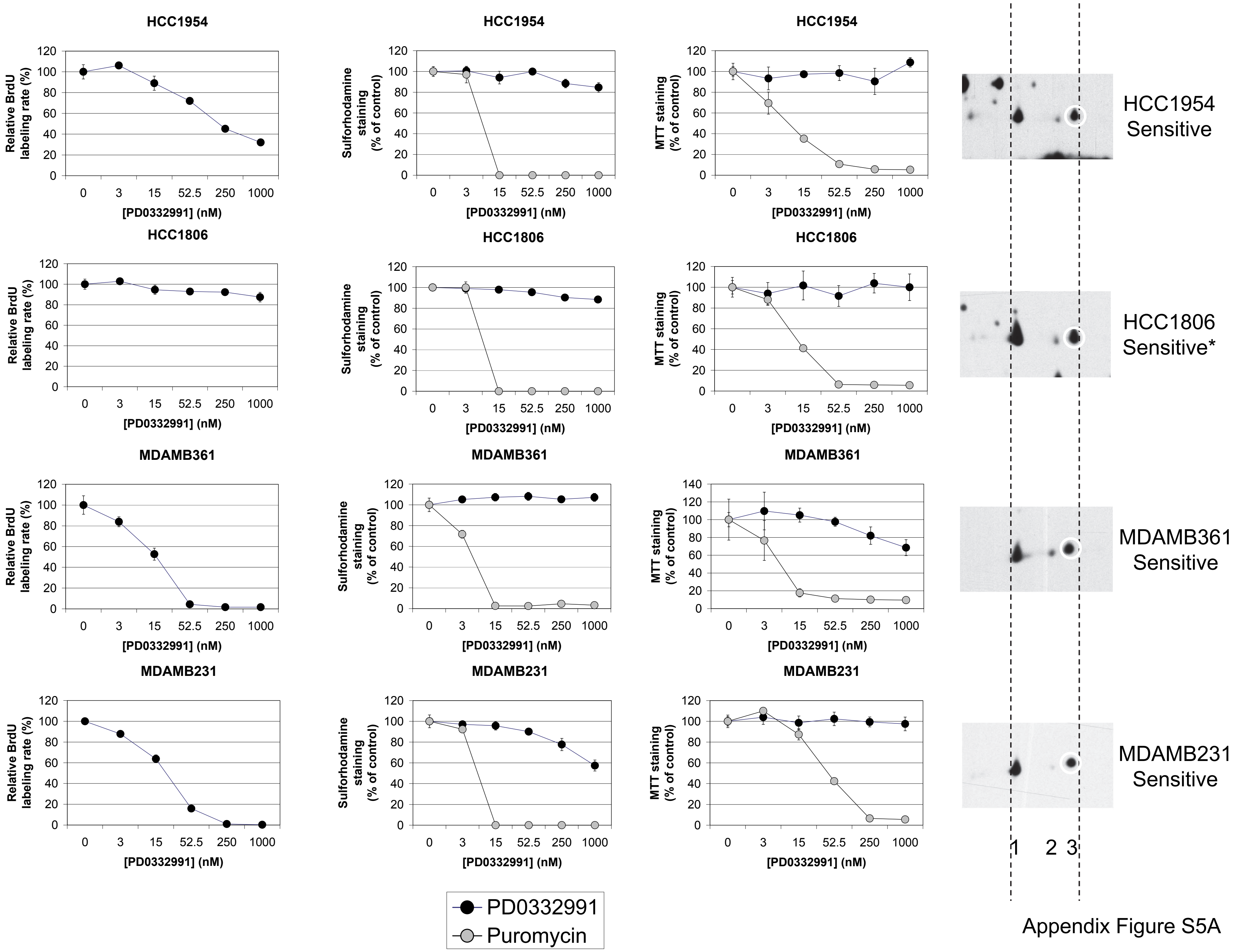

Appendix Figure S5A

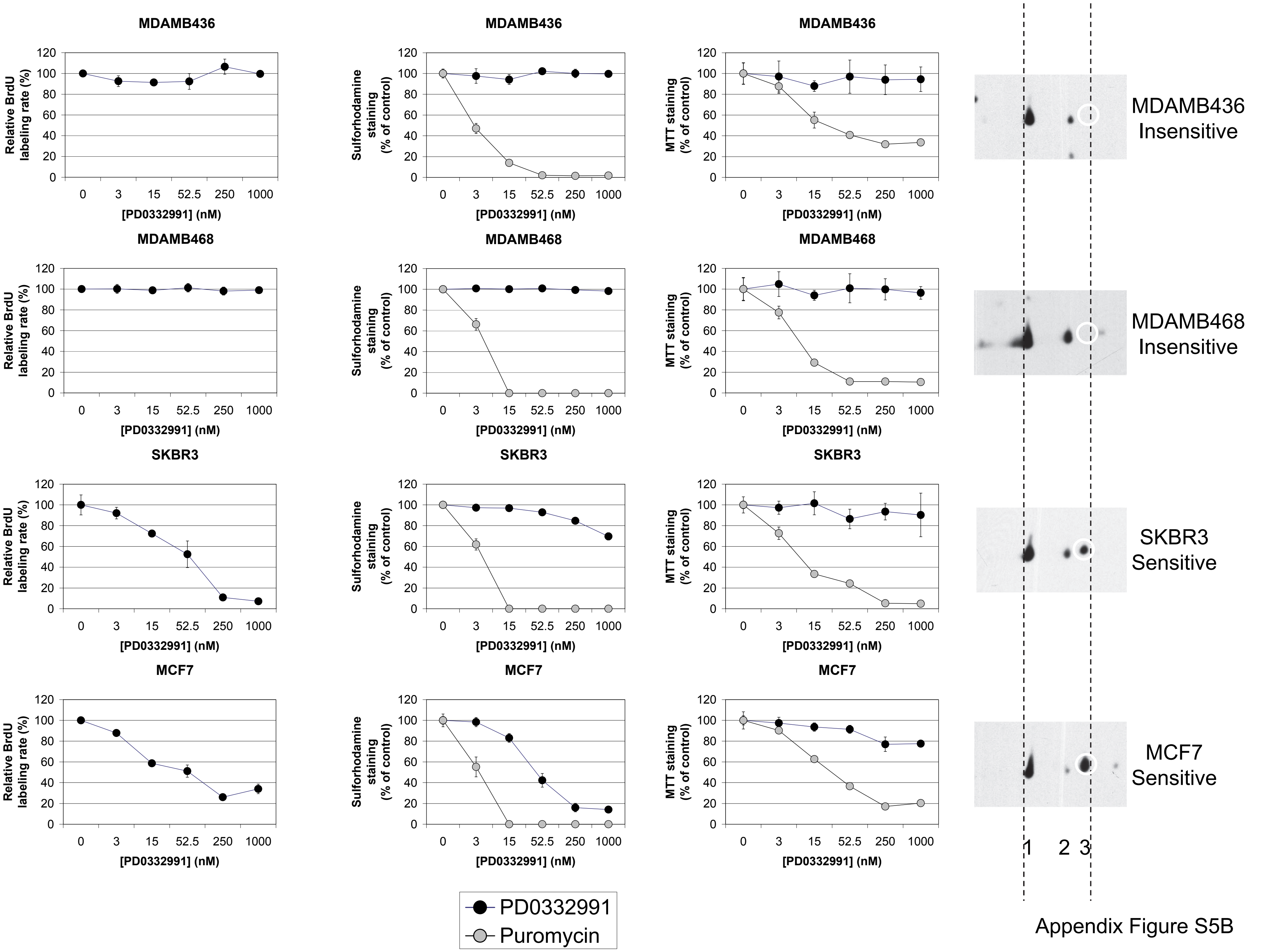

Appendix Figure S5B

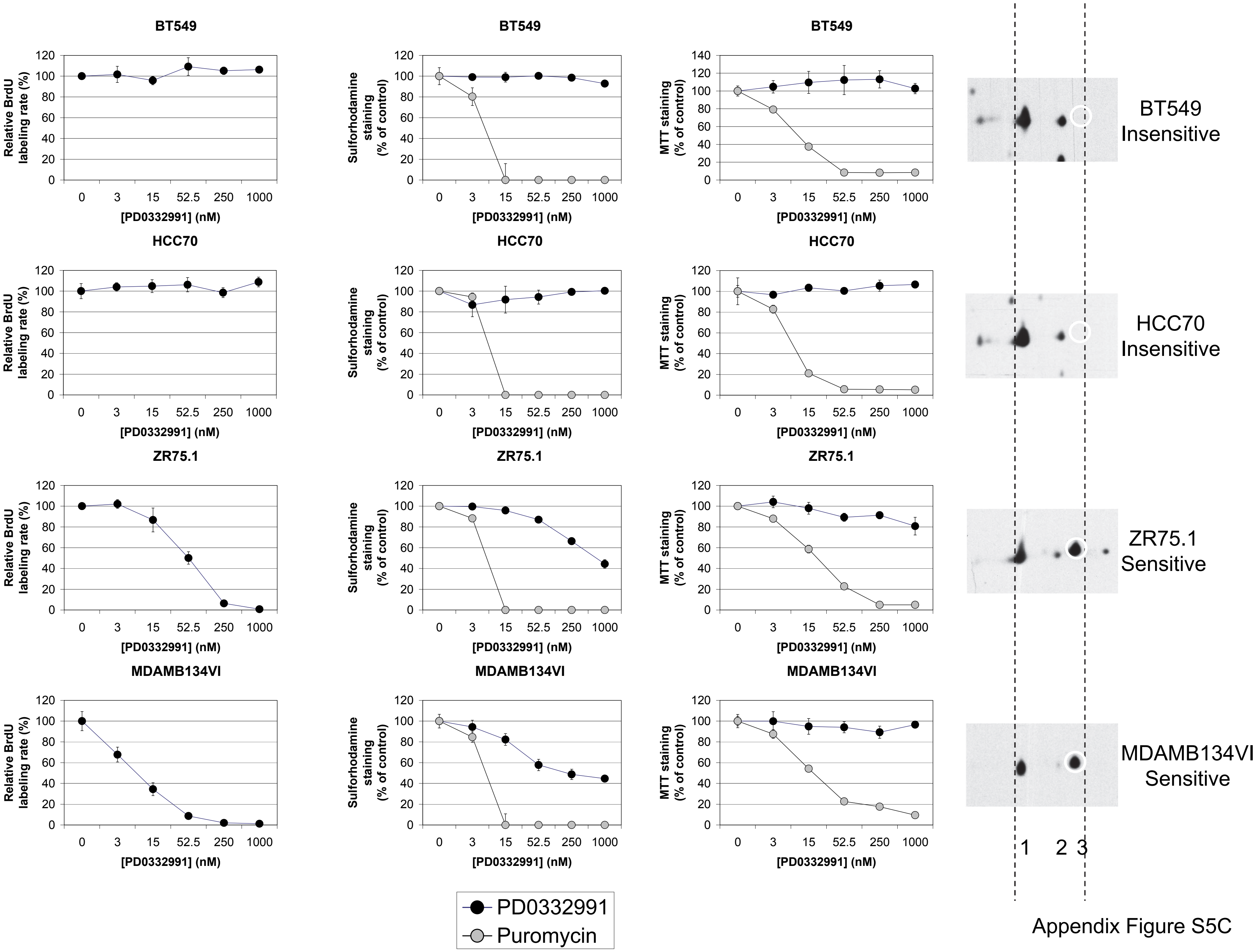

Appendix Figure S5C

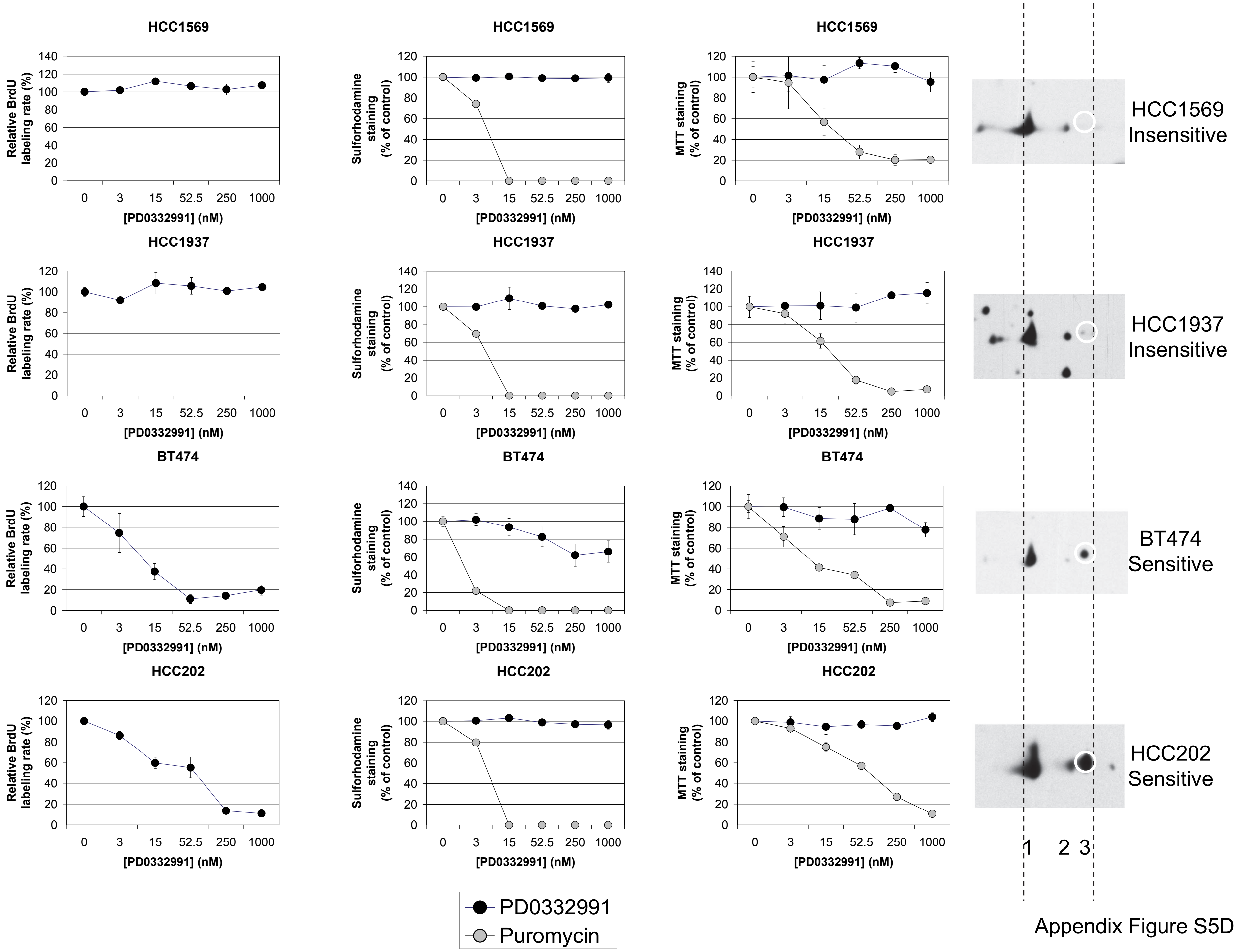

Appendix Figure S5D

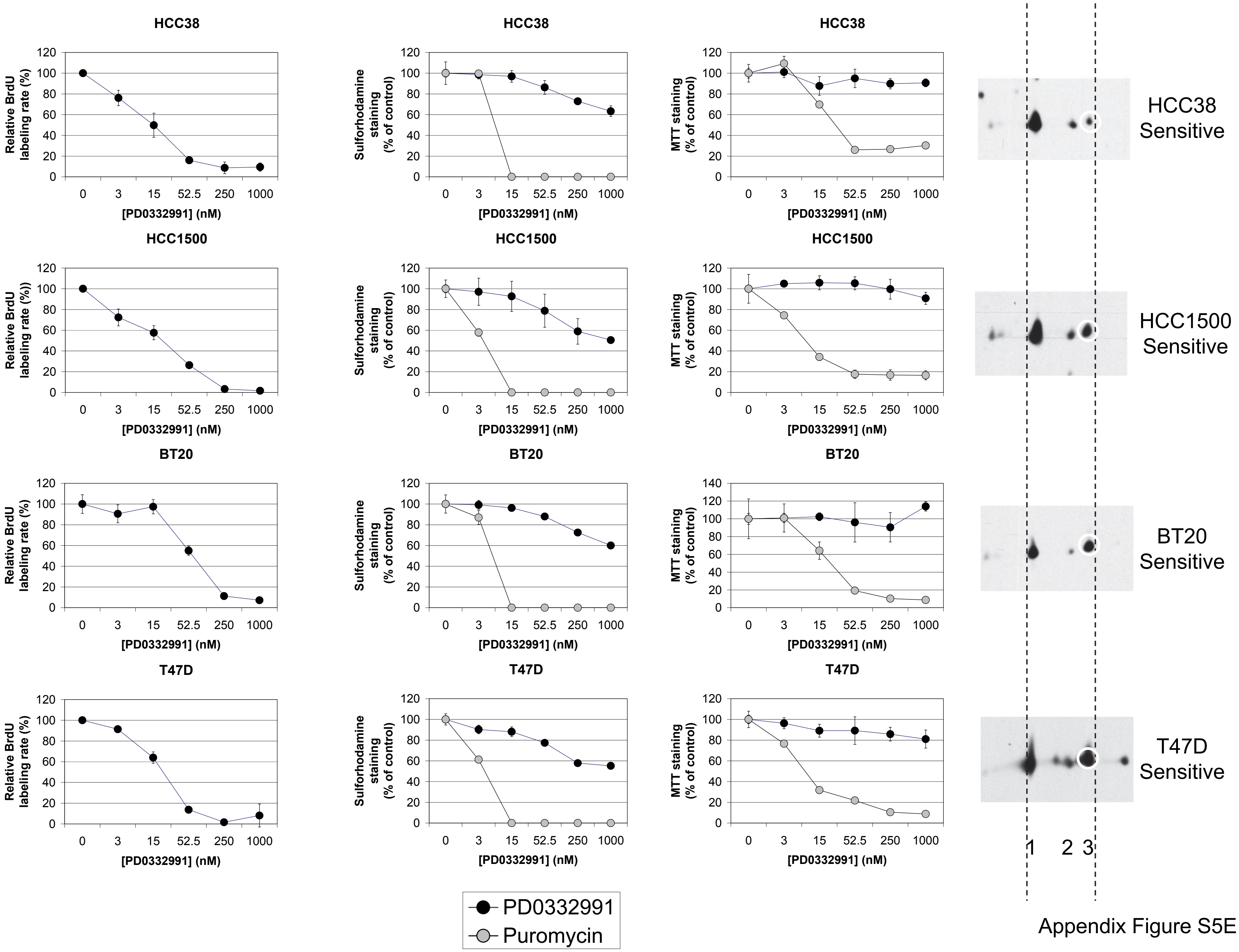

Appendix Figure S5E

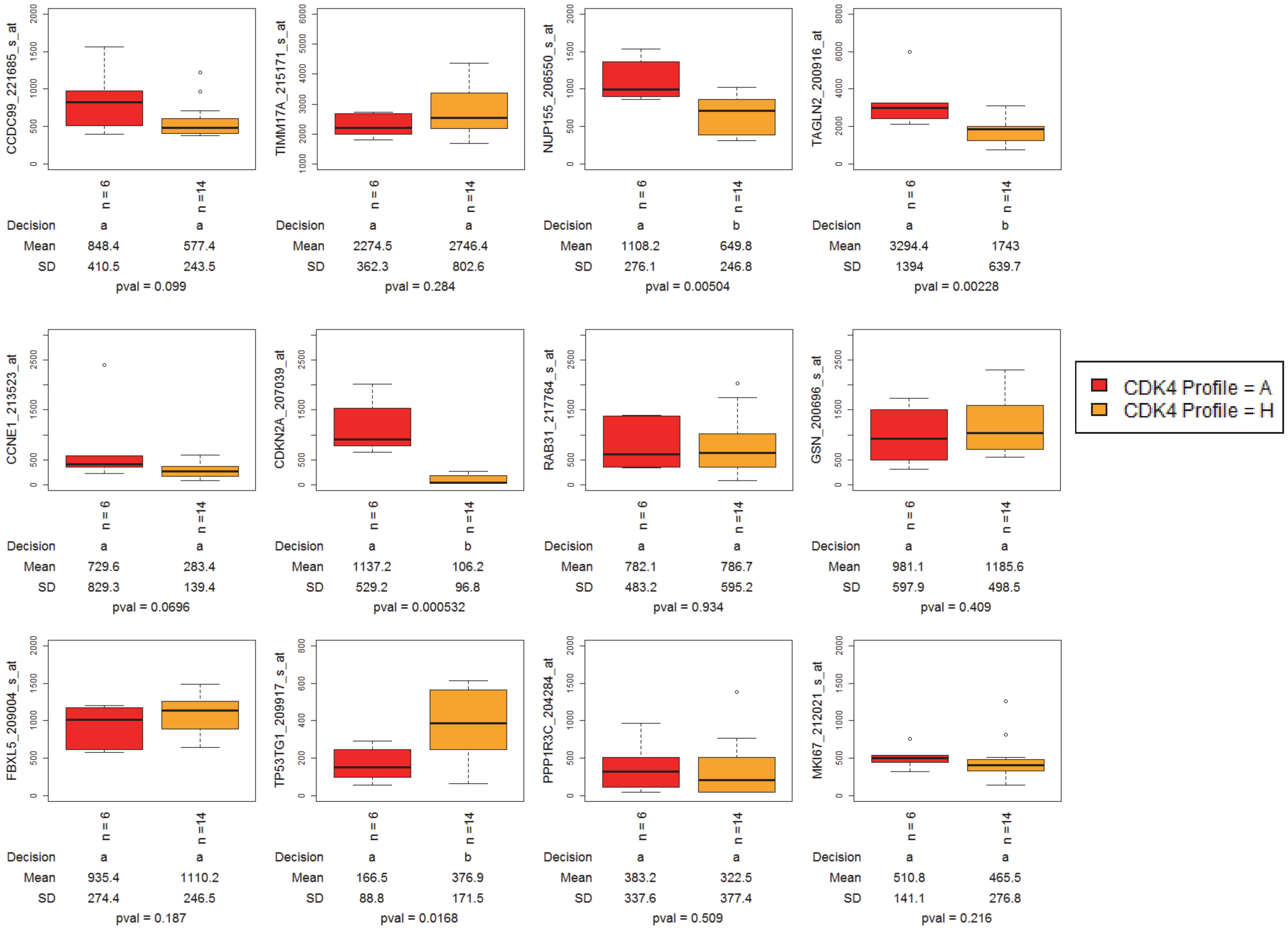

Appendix Figure S6

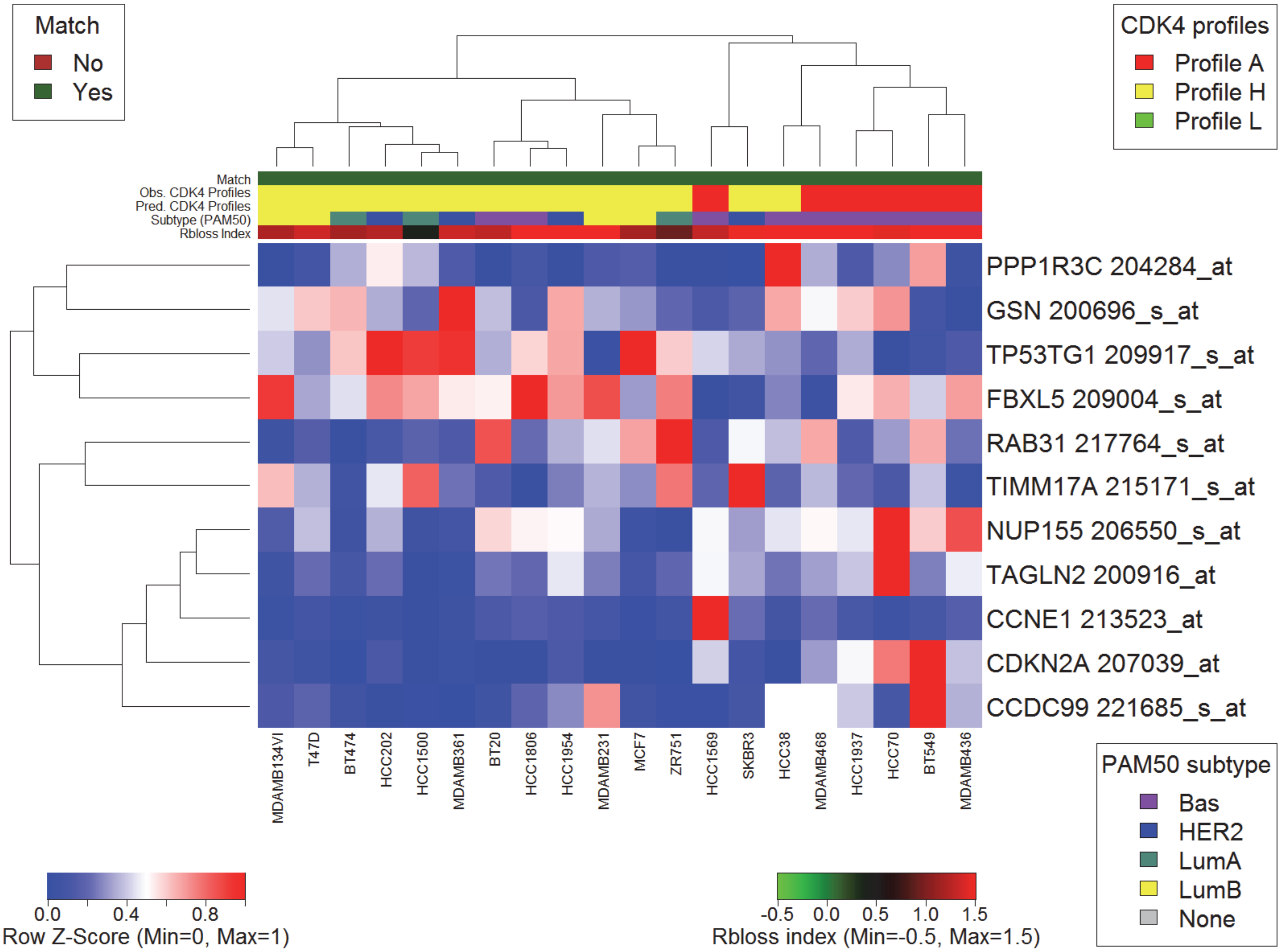

Appendix Figure S7

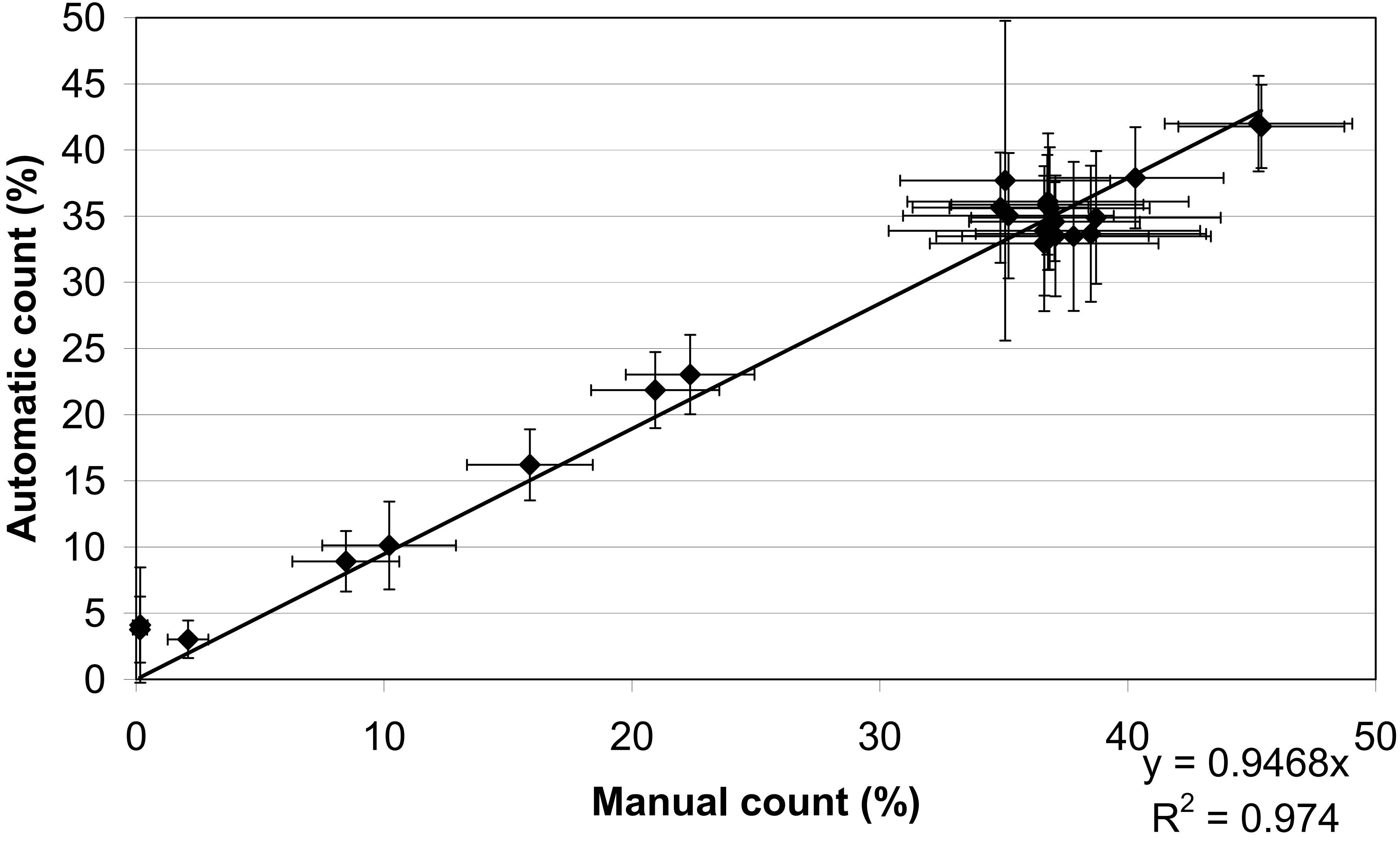

Appendix Figure S8

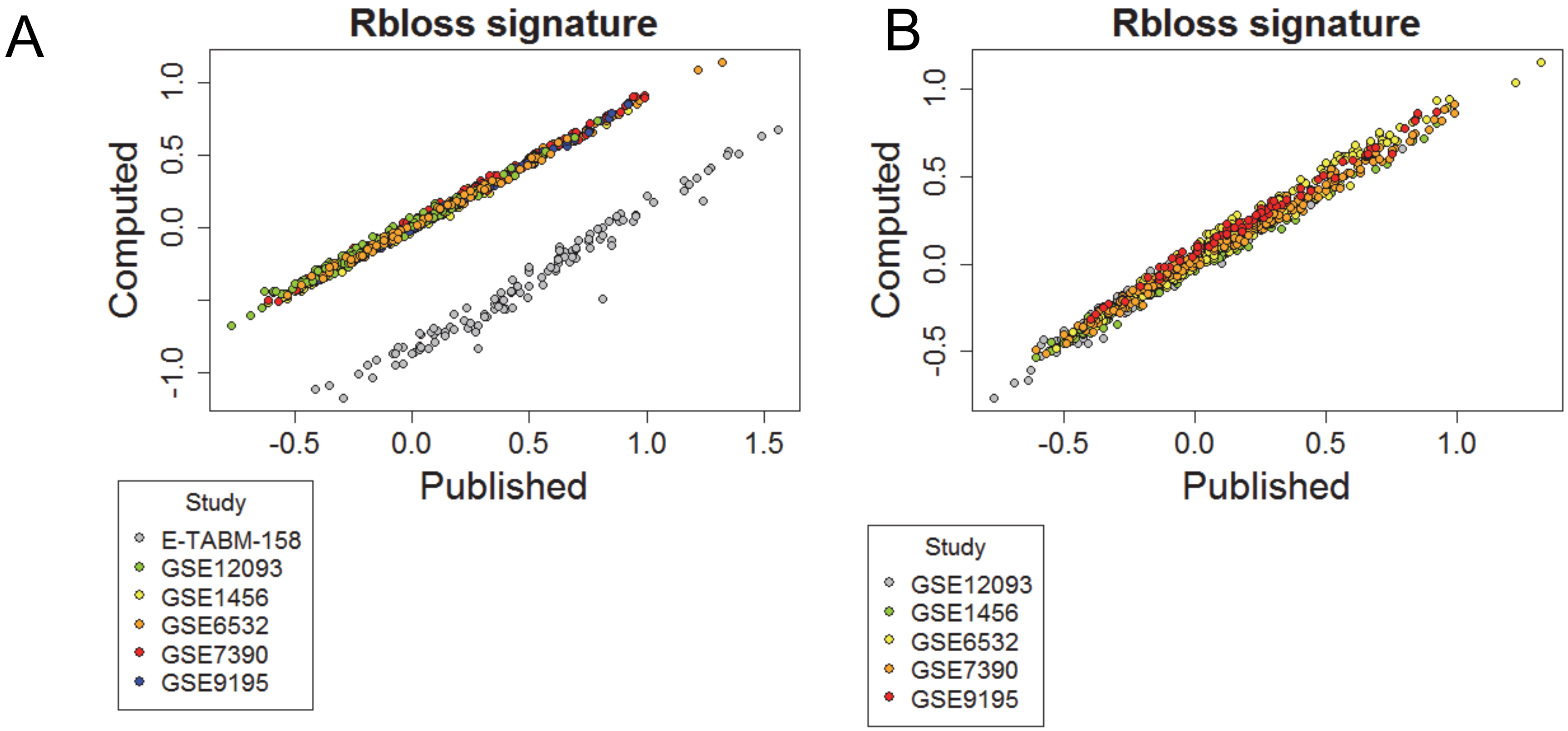

Appendix Figure S9

Antibodies and reagents	Provider	Reference	use	dilution
anti Ki-67	DAKO	IR626	IHC	1/1
anti BrDU	BD Bioscience	#347580	IHC	1/50
biotinylated anti-mouse	GE Healthcare	RPN1001V	IHC	1/50
Alexa Fluor 488-conjugated Streptavidin	Jackson ImmunoResearch	3016-480-084	IHC	1/3600
cyclin D1	Neomarkers	DCS-6	WB	1/200
cyclin D3	Neomarkers	DCS-22	WB	1/200
pRb	BD Pharmingen	#554136	WB	1/500
phospho-pRb (T826)	Abcam	EPR5351	WB	1/1000
cyclin E1	Pierce	HE12	WB	1/200
anti-CDK4 (monoclonal)	Santa Cruz	DCS-31	WB	1/200
anti-CDK4 (polyclonal)	Santa Cruz	H-22	WB	1/200
anti-P-CDK4 (polyclonal)	Cell Signaling Technology	*	WB	1/500
p16	Santa Cruz	DCS-50	WB	1/200
CDK6	Santa Cruz	C-21	WB	1/1000
HRP-coupled anti mouse	Santa Cruz	sc-2005	WB	1/2000
HRP-coupled anti rabbit	Santa Cruz	sc-2370	WB	1/5000
DyLight 680-coupled anti mouse	Pierce Biotechnology	#35518	WB	1/10000
DyLight 800-coupled anti rabbit	Pierce Biotechnology	#SA5-35571	WB	1/10000

* sample of a noncommercialized phosphospecific CDK4 (Thr172) antibody produced by immunizing rabbits with a keyhole limpet hemocyanin-coupled peptide antigen to T172-phosphorylated human CDK4 and purified by protein A- and immunogen-based affinity column separation. See Bockstaele et al Mol. Cell. Biol. 26,5070 (2006) for characterization. This antibody is very phosphospecific on CDK4 but generally requires a prior purification or separation of CDK4 (by IP and/or 2D electrophoresis). Nevertheless, it could be used (it was critical) in a study by Robert Fisher group (Merzel-Schachter et al Mol. Cell 50,250 (2013)). This preparation has never been commercialized and the bulk of its stock was unfortunately lost at CST. To our knowledge, CST was unable to reproduce it until now (one production was sold but it was inactive and removed from the catalog after 6 months). We preciously keep the few microliters left as reference material.

Appendix Supplementary Table S1

Appendix Supplementary Table S2

Study	Plat	Brand	Source	Author	GSE29288	GSE51086	GSE22821	GSE44552	GSE44836	GSE18496	GSE43567	GSE5846	GSE5720	ETABM157	GSE4342	GSE16795	GSE11812	GSE6569	GSE5845	MDACC	GSE36133	EMTAB37	GSE34211	GSE10843	GSE8332	GSE7127	GSE32474	GSE10890	GSE10916	GSE12790	GSE22183	GSE15455	GSE41445	GSE3156	GSE4536
GSE29288	GPL4133	Agilent	NCI60	Reinhold	60	4	59	5	3	6	2	59	58	6	2	4	1	4	0	5	37	16	40	20	15	2	58	6	0	4	0	0	12	5	1
GSE51086	GPL4133	Agilent	Breast	Cunliffe	4	29	4	22	16	22	0	4	4	24	0	22	2	12	0	15	22	6	18	11	0	0	4	18	0	21	0	0	4	12	0
GSE22821	GPL4868	Agilent	NCI60	Liu	59	4	60	5	3	6	2	58	57	6	2	4	1	4	0	5	36	15	39	20	14	2	57	6	0	4	0	0	12	5	1
GSE44552	GPL6480	Agilent	Breast	Finn	5	22	5	52	24	48	0	5	5	34	0	23	2	17	0	14	41	18	33	22	1	0	5	35	0	37	0	0	4	15	0
GSE44836	GPL6480	Agilent	Breast	Di Cello	3	16	3	24	26	24	0	3	3	23	0	18	2	13	0	11	24	9	21	14	0	0	3	21	0	23	0	0	3	11	0
GSE18496	GPL7264	Agilent	Breast	Finn	6	22	6	48	24	51	0	6	6	35	0	23	2	18	0	14	40	18	34	21	1	0	6	35	0	36	0	0	4	16	0
GSE43567	GPL7264	Agilent	NSCL	Finn	2	0	2	0	0	0	34	1	0	0	7	0	0	0	0	0	23	15	9	16	13	0	2	0	0	0	0	0	1	0	0
GSE5846	GPL96	Affymetrix	NCI60	Lee	59	4	58	5	3	6	1	60	59	6	1	4	1	4	0	5	36	16	39	19	14	2	57	6	0	4	0	0	11	5	1
GSE5720	GPL96	Affymetrix	NCI60	Shankavaram	58	4	57	5	3	6	0	59	59	6	1	4	1	4	0	5	35	15	38	18	13	2	56	6	0	4	0	0	11	5	1
ETABM157	GPL96	Affymetrix	Breast	Neve	6	24	6	34	23	35	0	6	6	51	0	27	2	18	0	17	32	11	32	17	1	0	6	28	0	29	0	0	4	17	0
GSE4342	GPL96	Affymetrix	NSCLC	Coldren	2	0	2	0	0	0	7	1	1	0	41	0	0	0	0	0	26	14	13	4	11	0	2	0	0	0	0	0	2	0	0
GSE16795	GPL96	Affymetrix	Breast	Holestelle	4	22	4	23	18	23	0	4	4	27	0	39	2	12	0	15	24	9	20	13	0	0	4	19	0	25	0	0	3	13	0
GSE11812	GPL96	Affymetrix	Multi	Gyorffy	1	2	1	2	2	2	0	1	1	2	0	2	30	2	0	2	12	8	2	6	1	0	1	1	1	2	0	0	1	2	0
GSE6569	GPL96	Affymetrix	Breast	Huang	4	12	4	17	13	18	0	4	4	18	0	12	2	23	0	12	20	6	17	12	0	0	4	17	0	18	0	0	2	14	0
GSE5845	GPL96	Affymetrix	Bladder	Lee	0	0	0	0	0	0	0	0	0	0	0	0	0	0	40	0	0	0	0	0	0	0	0	0	0	0	0	0	0	0	0
MDACC	GPL96	Affymetrix	Breast	MDACC	5	15	5	14	11	14	0	5	5	17	0	15	2	12	0	19	16	4	13	11	1	0	5	14	0	15	0	0	3	12	0
GSE36133	GPL570	Affymetrix	Multi	CCLE	37	22	36	41	24	40	23	36	35	32	26	24	12	20	0	16	916	236	109	80	87	5	36	40	1	45	15	12	14	18	2
EMTAB37	GPL570	Affymetrix	Multi	GSK	16	6	15	18	9	18	15	16	15	11	14	9	8	6	0	4	236	319	46	35	46	0	16	16	1	16	4	4	3	6	2
GSE34211	GPL570	Affymetrix	Multi	Pfizer	40	18	39	33	21	34	9	39	38	32	13	20	2	17	0	13	109	46	166	35	24	2	40	30	0	30	13	11	10	15	1
GSE10843	GPL570	Affymetrix	Multi	Adai	20	11	20	22	14	21	16	19	18	17	4	13	6	12	0	11	80	35	35	134	48	3	20	26	2	27	0	0	10	11	0
GSE8332	GPL570	Affymetrix	Multi	Wagner	15	0	14	1	0	1	13	14	13	1	11	0	1	0	0	1	87	46	24	48	119	3	15	1	2	0	0	0	6	0	0
GSE7127	GPL570	Affymetrix	Melanoma	Johansson	2	0	2	0	0	0	0	2	2	0	0	0	0	0	0	0	5	0	2	3	3	63	2	0	1	0	0	0	1	0	0
GSE32474	GPL570	Affymetrix	NCI60	Pfister	58	4	57	5	3	6	2	57	56	6	2	4	1	4	0	5	36	16	40	20	15	2	59	6	0	4	0	0	11	5	1
GSE10890	GPL570	Affymetrix	Breast	Hoeflich	6	18	6	35	21	35	0	6	6	28	0	19	1	17	0	14	40	16	30	26	1	0	6	55	0	41	0	0	4	16	0
GSE10916	GPL570	Affymetrix	Melanoma	Augustine	0	0	0	0	0	0	0	0	0	0	0	0	1	0	0	0	1	1	0	2	2	1	0	0	50	0	0	0	0	0	0
GSE12790	GPL570	Affymetrix	Breast	Januario	4	21	4	37	23	36	0	4	4	29	0	25	2	18	0	15	45	16	30	27	0	0	4	41	0	50	0	0	4	16	0
GSE22183	GPL570	Affymetrix	Stomach	Anderson	0	0	0	0	0	0	0	0	0	0	0	0	0	0	0	0	15	4	13	0	0	0	0	0	0	0	37	23	0	0	0
GSE15455	GPL570	Affymetrix	Stomach	Ooi	0	0	0	0	0	0	0	0	0	0	0	0	0	0	0	0	12	4	11	0	0	0	0	0	0	0	23	25	0	0	0
GSE41445	GPL570	Affymetrix	Multi	Bayer	12	4	12	4	3	4	1	11	11	4	2	3	1	2	0	3	14	3	10	10	6	1	11	4	0	4	0	0	21	3	0
GSE3156	GPL570	Affymetrix	Breast	Bild	5	12	5	15	11	16	0	5	5	17	0	13	2	14	0	12	18	6	15	11	0	0	5	16	0	16	0	0	3	19	0
GSE4536	GPL570	Affymetrix	Glioblastoma	Fine	1	0	1	0	0	0	0	1	1	0	0	0	0	0	0	0	2	2	1	0	0	0	1	0	0	0	0	0	0	0	12
