# Supplementary material for: CDK4 phosphorylation status and a linked gene expression profile predict sensitivity to palbociclib
Source: EMBO Mol Med. 2017 May 31;9(8):1052–66. doi: 10.15252/emmm.201607084 (PMC5538335; doi:10.15252/emmm.201607084)
Supplement: Supplementary file 2 — Expanded View Figures PDF [file EMMM-9-1052-s002.pdf]

Expanded View Figures

Figure EV1. Observed and predicted CDK4 modification profiles differentially associate with different clinical parameters in the 56 breast tumors examined in this study and in a cohort of 4,034 tumors with published gene expression profiles.

The relative proportions of the observed CDK4 modification profiles according to the different levels of the ER, PR, HER2, and triple-negative (TNM) status, intrinsic molecular subtypes, grade, GGI risk, and Oncotype DX risk of the 56 breast tumors samples analyzed in this study (topmost panels) are compared to the relative proportions of the predicted values of the same samples (middle panels) and those predicted using a database of 4,034 published gene expression profiles of breast cancer samples with published clinical records (lowest panels).

A The ER, PR, and HER2 statuses were defined by immunohistochemistry. The triple-negative status was defined based on whether the gene expression of *ESR1*, *PGR*, and *ERBB2* genes measured using the probe sets 205225_at, 208305_at, and 216836_s_at, respectively, were below a threshold predefined with pROC.

B The molecular subtype, grade, GGI risk, and Oncotype DX risk (OnDx) were determined in R with the genefu package using the corresponding reference probe sets.

 © 2017 The Authors

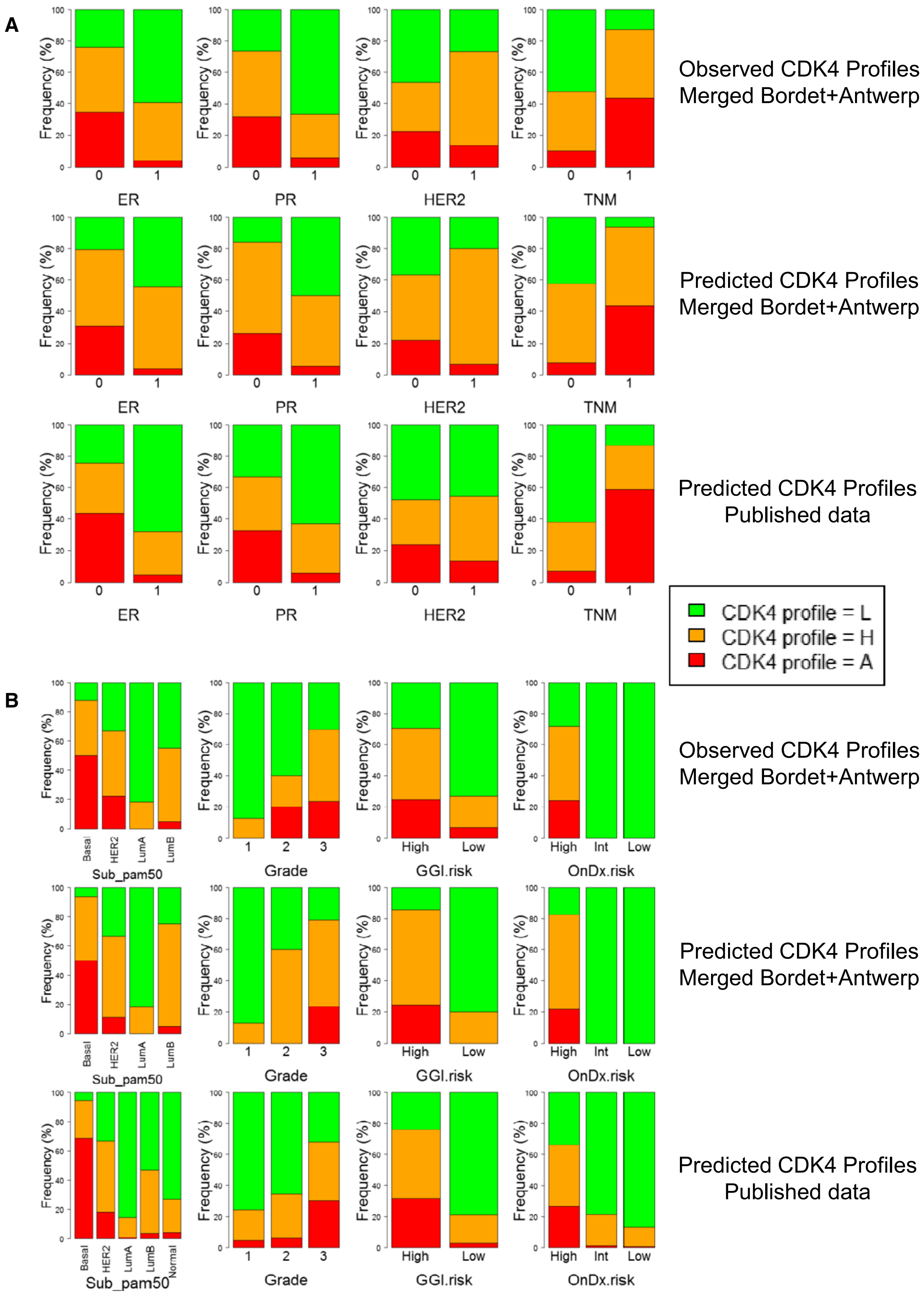
A
B
Frequency (%)
ER
PR
HER2
TNM
Observed CDK4 Profiles
Merged Bordet+Antwerp
Predicted CDK4 Profiles
Merged Bordet+Antwerp
Predicted CDK4 Profiles
Published data
CDK4 profile = L
CDK4 profile = H
CDK4 profile = A
Basal
HER2
LumA
LumB
Normal
Sub_pam50
Grade
High
Low
GGI.risk
Int
OnDx.risk

Figure EV1.

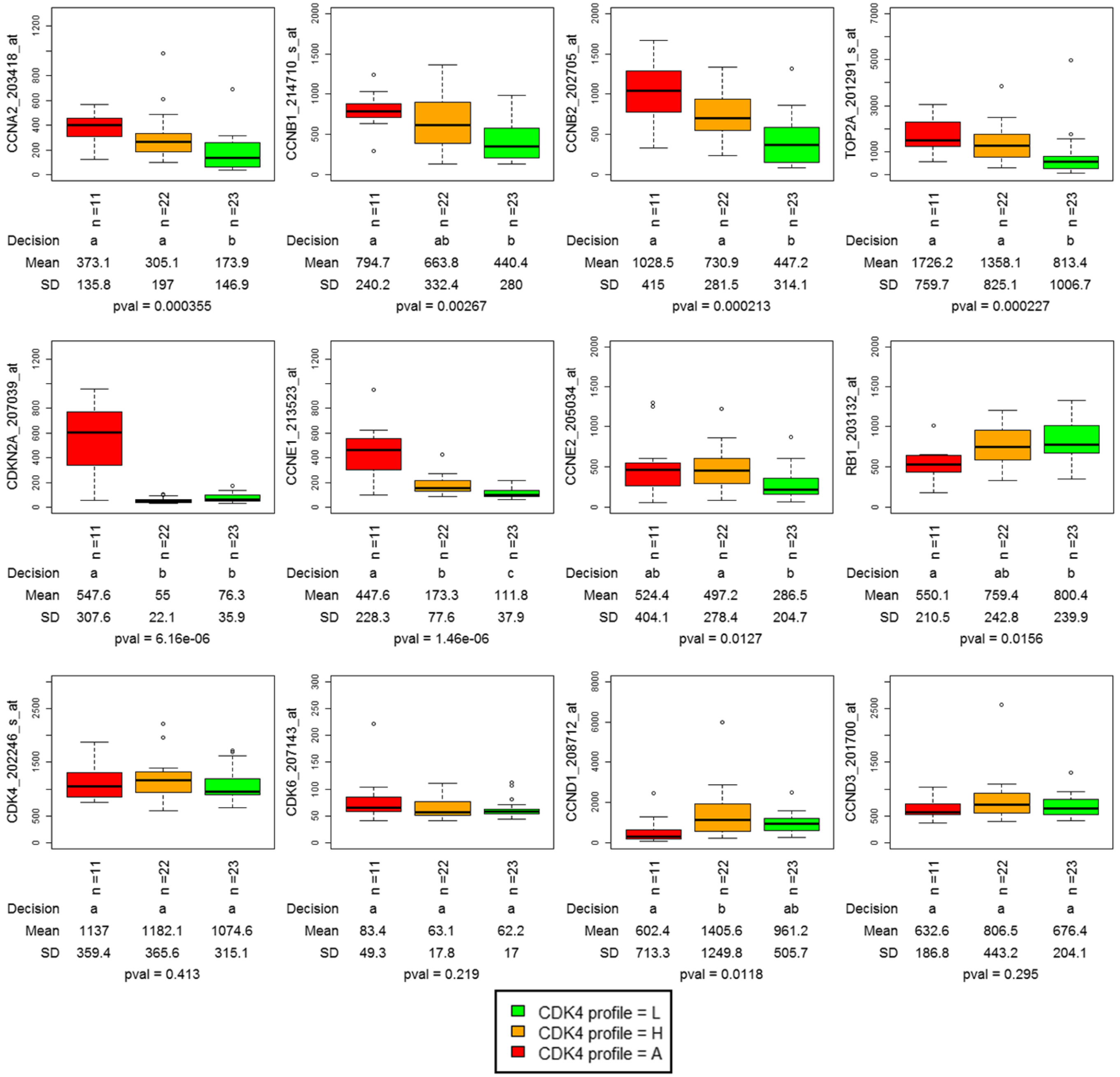

Figure EV2. Distributions of expression levels of selected cell cycle genes among tumors with the three observed CDK4 modification profiles.
The gene expression levels of selected cell cycles genes were measured using the indicated probe sets. The distributions of these levels among the three CDK4 modification profiles are illustrated. Only the expression levels of *CDKN2A*, *CCNE1*, and *CCND1* were significantly different in profile A relative to profile H tumors. By contrast, expression levels of *CCNA2*, *CCNB1*, *CCNB2*, *TOP2A*, and *CCNE2* were significantly lower in profile L tumors than in profile A and/or profile H tumors. Data (box and whiskers) represent median, quartiles and the largest and smallest values with outliers excepted. The first line below the plot reports whether the true effect of CDK4 modification profile is significant (levels with the same letter are not significantly different at $\alpha = 0.05$). The second and third lines report the respective means and SD. The last line provides the *P*-value of rejection of the null hypothesis that all means are equal. Pairwise comparisons were performed with the Kruskal–Wallis test (level of confidence set at 0.95).

 © 2017 The Authors

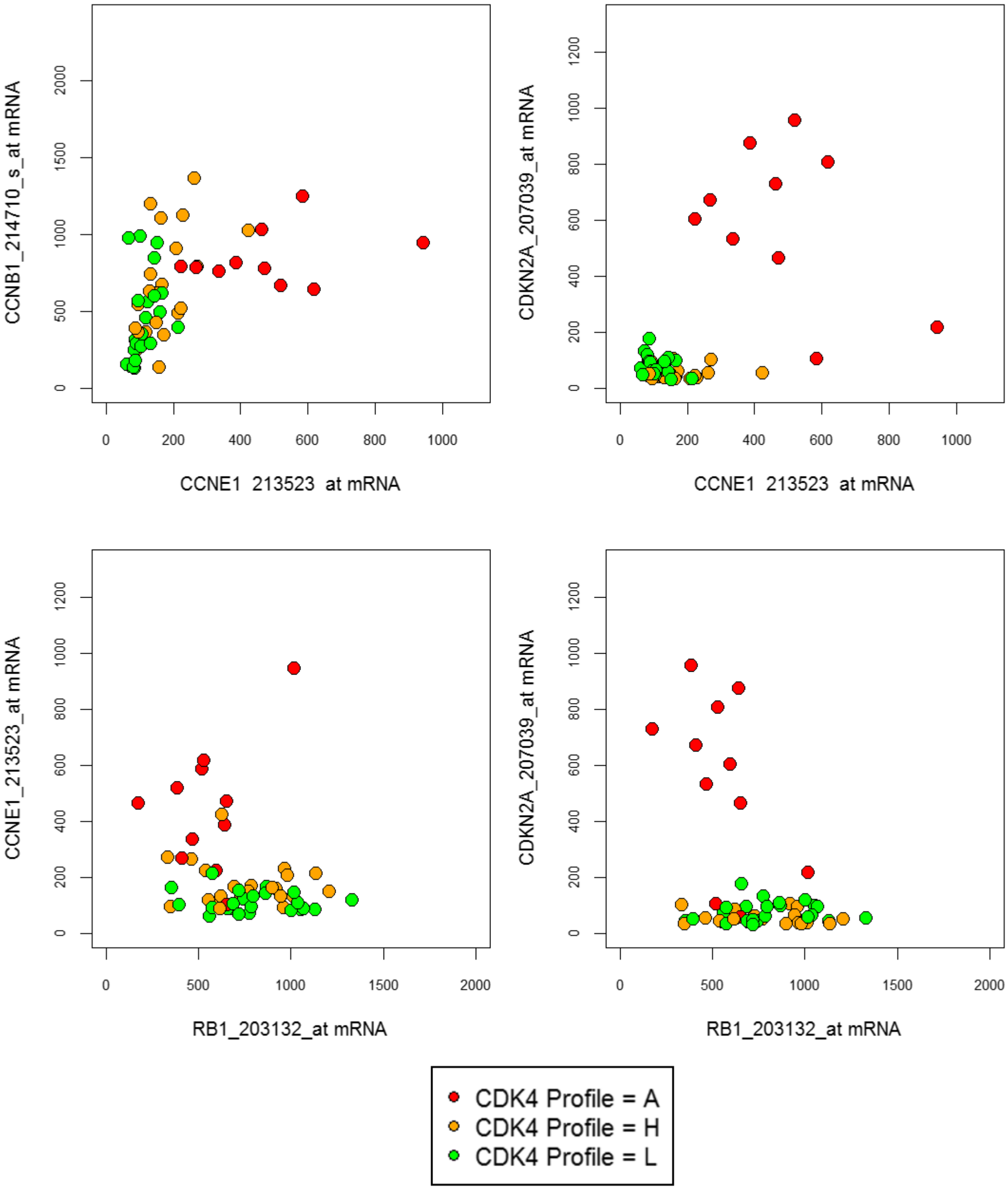

Figure EV3. Relationships among selected cell cycle markers in breast tumors according to their CDK4 modification profiles.
The gene expression levels of selected cell cycles genes were measured with the Affymetrix HG-U133 plus2 platform using the indicated probe sets. The following comparisons are illustrated for tumors characterized by the three CDK4 modification profiles: *CCNE1* and *CCNB1* (cyclin B1) used as a cell cycle progression marker; *CCNE1* and *CDKN2A*; *RB1* and *CCNE1*; *RB1* and *CDKN2A*.

© 2017 The Authors

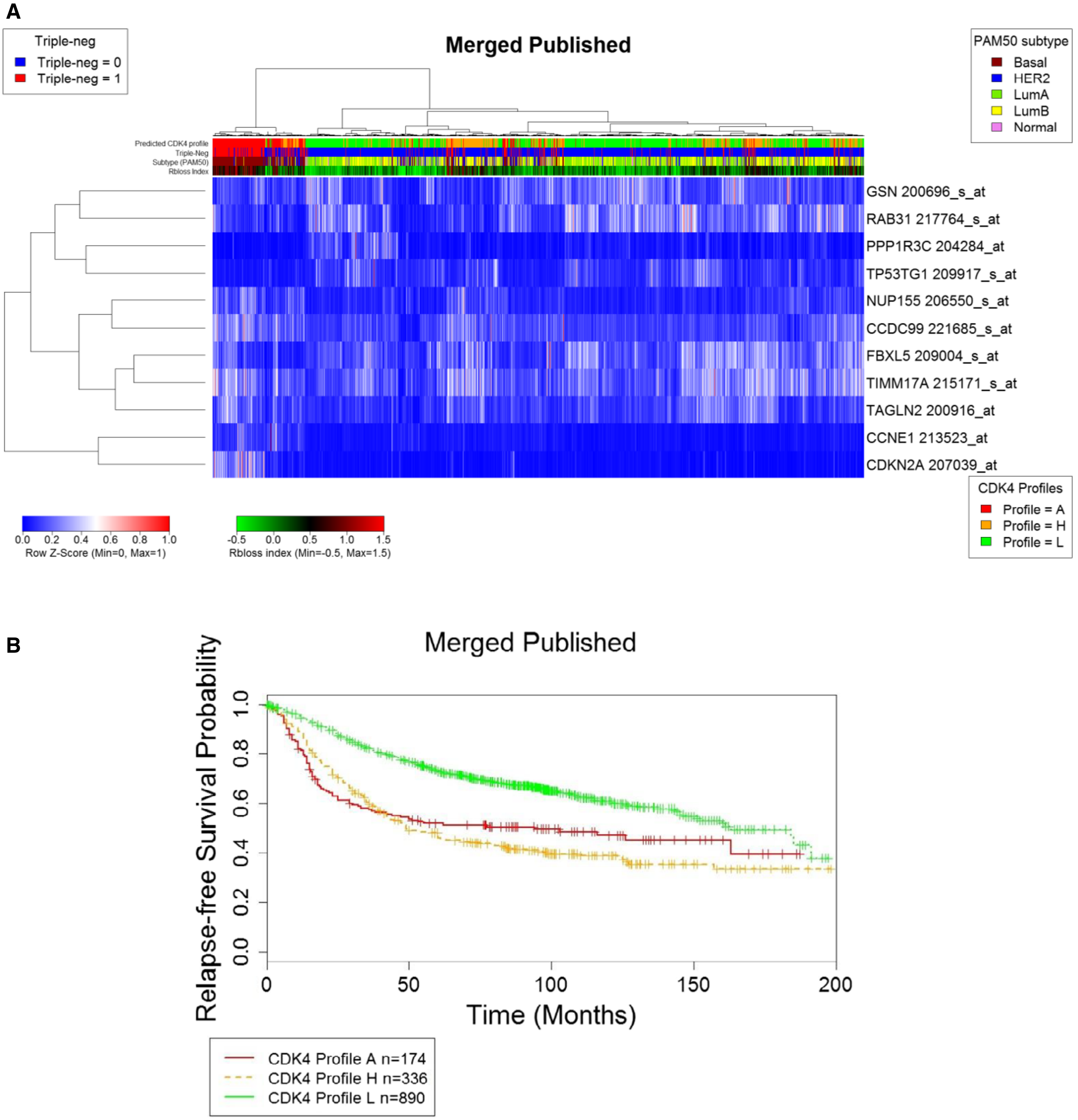

Figure EV4. The 11-probe predictor of CDK4 modification profiles classifies 4,034 breast tumors and is associated with their relapse hazard.

A The published expression levels of the 11 selected genes (see Dataset EV7 for the probes) of the CDK4 modification profile predictor are displayed for 4,034 tumors analyzed with the Affymetrix HG-U133 plus2 or HG-U133A platforms. The heatmap was drawn with the heatmap.plus R package using the fRMA-normalized expression values of the 11 probes. Displayed above the heatmap are the predicted CDK4 modification profiles, the triple-negative status, the molecular subtype defined with the genefu package by the PAM50 expression value, and the Rb loss index computed as reported by Knudsen's laboratory (Ertel *et al*, 2010).

B The association of the predicted CDK4 modification profile type with relapse hazard was estimated using the Survival package in R in a cohort of 1,400 non-redundant patients with available survival data and gene expression profiles published before September 2009.

 © 2017 The Authors

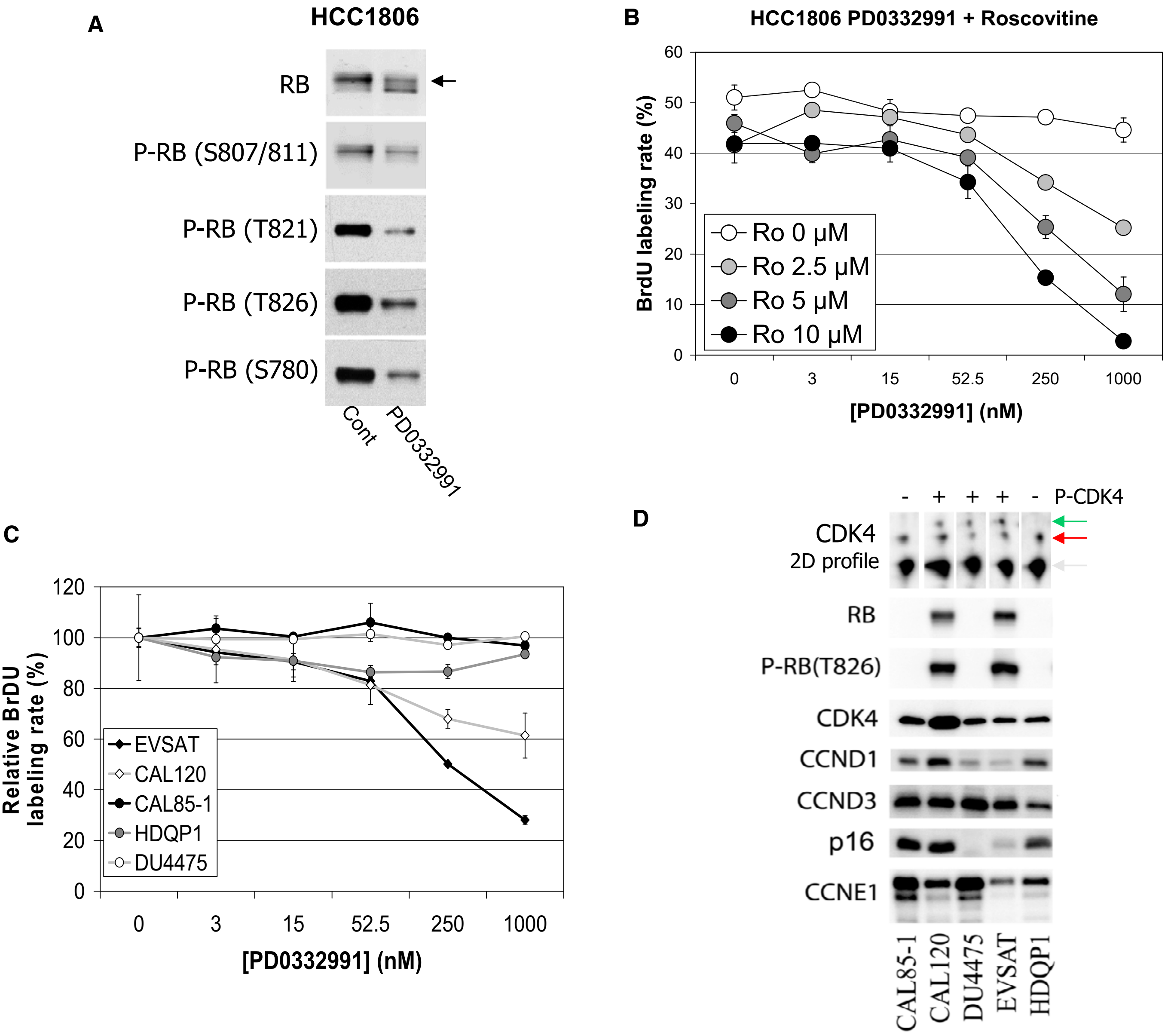

Figure EV5. Characterization of PD0332991 sensitivity and proteomic profiles of HCC1806, CAL85-1, CAL120, DU4475, EVSAT, and HDQP1 breast cancer cell lines.

A Effect of PD0332991 on different phosphorylations of pRb. HCC1806 cells were cultured in the presence of 10% FBS and challenged with or without (Cont) 250 nM PD0332991 for 24 h. pRb and its phosphorylations were immunodetected after SDS–PAGE of whole-cell extracts. The arrow indicates the band corresponding to hyperphosphorylated pRb.

B Effects of the combined administration of increasing concentrations of PD0332991 and R-roscovitine (Ro) on the proportion of BrdU-incorporating HCC1806 cells. Treatment and BrdU labeling are as in Fig 4. Values are mean ± SD from triplicates.

C Effect of increasing concentrations of PD0332991 on the DNA synthesis rate of CAL85-1, CAL120, DU4475, EVSAT, and HDQP1 breast cancer cell lines. Treatment and BrdU labeling are as in Fig 4. Relative proportion of BrdU-labeled cells is expressed as % of the mean value of untreated control cells. Values are mean ± SD from triplicates.

D CDK4 modification profile and expression of cell cycle regulatory proteins in CAL85-1, CAL120, DU4475, EVSAT, and HDQP1 breast cancer cell lines. Total protein extracts from asynchronous cells in culture were resolved by SDS–PAGE or 2D-gel electrophoresis and detected with the indicated antibodies. The position of the T172-phosphorylated form of CDK4 is indicated by a green arrow. Grey and red arrows indicate the non-phosphorylated forms of CDK4 corresponding to spots 1 & 2, respectively. Chemiluminescence was imaged with a Vilber Lourmat Fusion Solo camera.

© 2017 The Authors
