# Supplementary material for: CDK4 phosphorylation status and a linked gene expression profile predict sensitivity to palbociclib
Source: EMBO Mol Med. 2017 May 31;9(8):1052–66. doi: 10.15252/emmm.201607084 (PMC5538335; doi:10.15252/emmm.201607084)
Supplement: Supplementary file 13 — Source Data for Figure 5 [file EMMM-9-1052-s012.pdf]

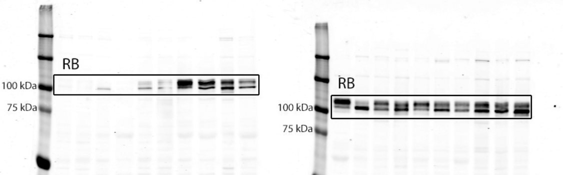
RB
100 kDa
75 kDa
RB
100 kDa
75 kDa

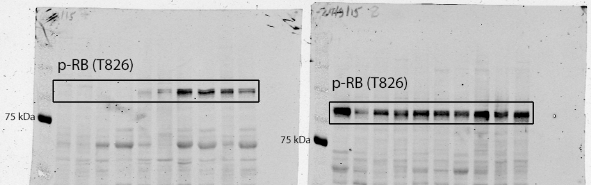

p-RB (T826)
75 kDa
p-RB (T826)
75 kDa

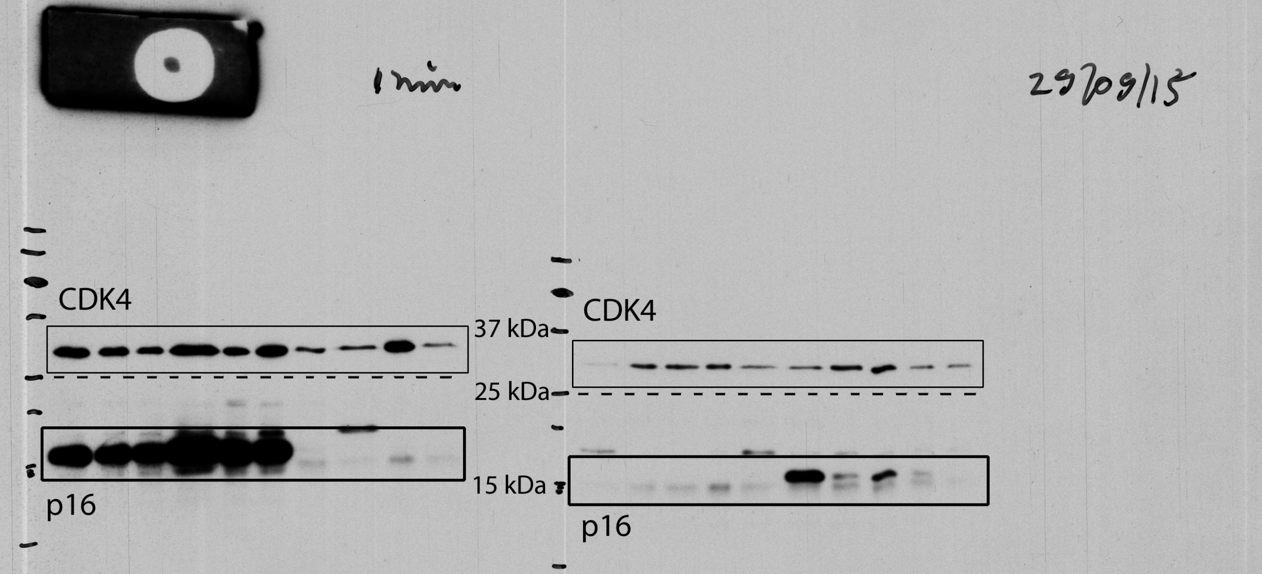

<u>Note:</u> For detections of CDK4 and p16, the membranes were cut at molecular weight 25 kDa (broken line).
CDK4 and p16 antibodies were incubated separetely.
Membranes were reassembled for film exposure.

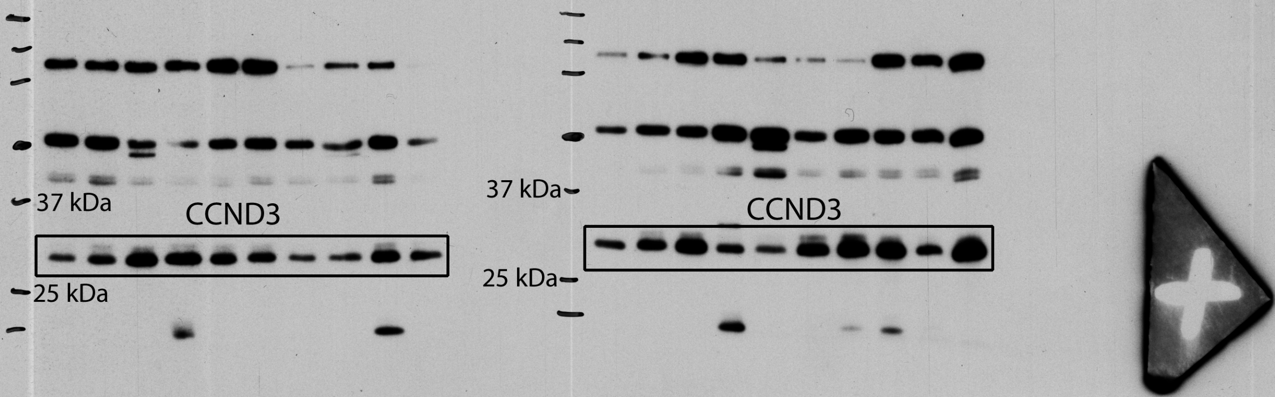

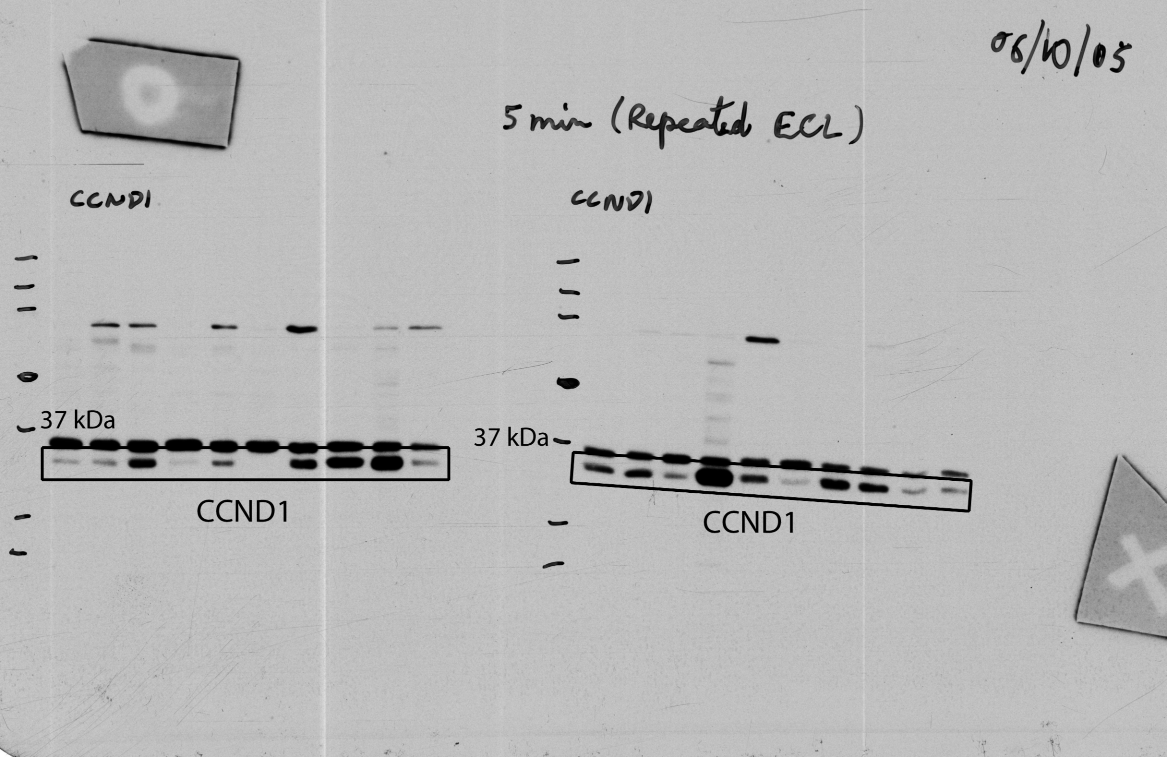
06/10/05
5 min (Repeated ECL)
CCND1
37 kDa
CCND1
CCND1
37 kDa
CCND1

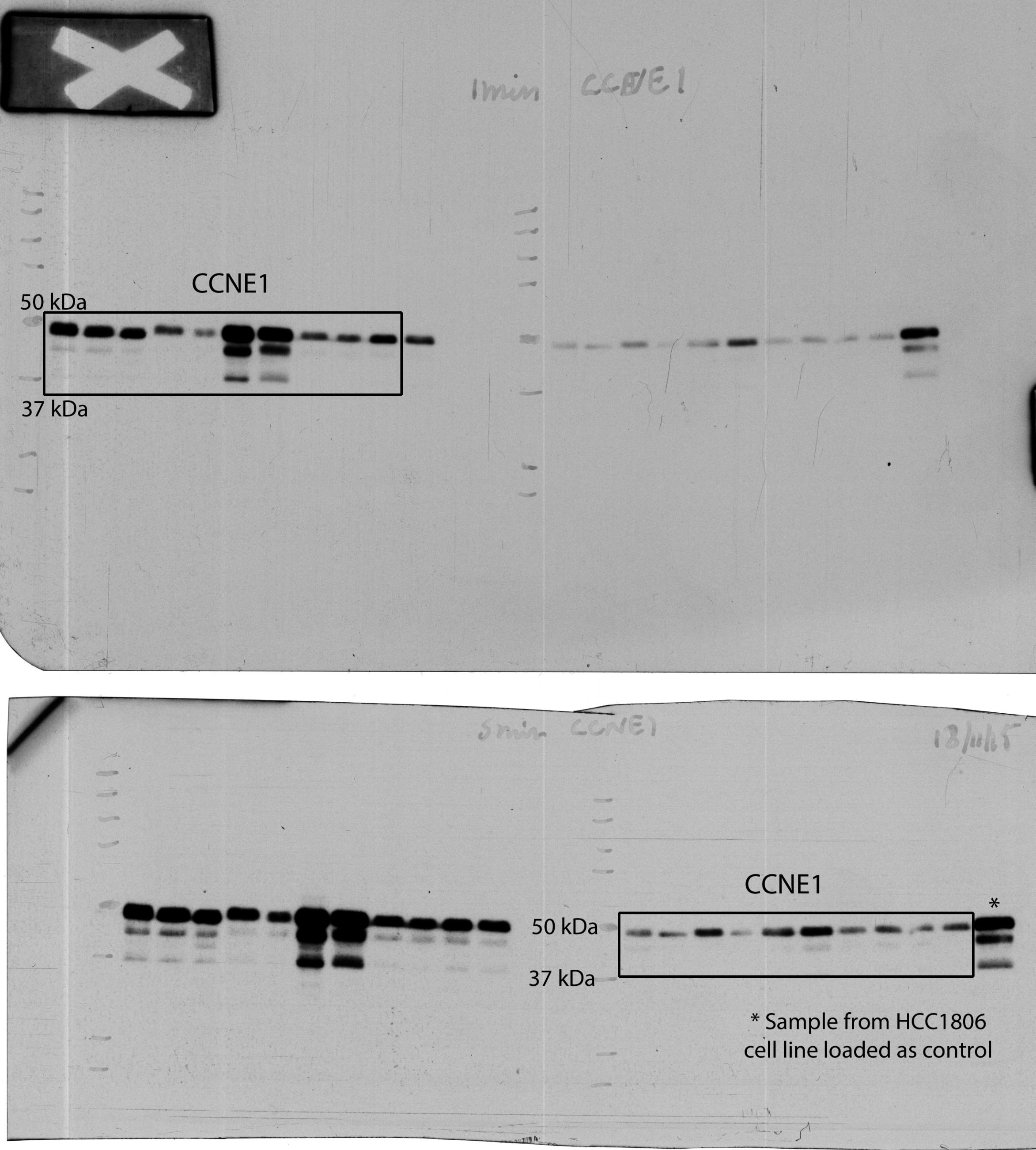
1min CCNE1
CCNE1
50 kDa
37 kDa
5min CCNE1
18/11/15
CCNE1
50 kDa
37 kDa
*
* Sample from HCC1806 cell line loaded as control
